# Entomopathogenic pseudomonads can share an insect host with entomopathogenic nematodes and their mutualistic bacteria

**DOI:** 10.1093/ismejo/wrae028

**Published:** 2024-02-21

**Authors:** Maria Zwyssig, Anna Spescha, Tabea Patt, Adrian Belosevic, Ricardo A R Machado, Alice Regaiolo, Christoph Keel, Monika Maurhofer

**Affiliations:** Plant Pathology, Institute of Integrative Biology, Swiss Federal Institute of Technology (ETH) Zurich, CH-8092 Zurich, Switzerland; Plant Pathology, Institute of Integrative Biology, Swiss Federal Institute of Technology (ETH) Zurich, CH-8092 Zurich, Switzerland; Plant Pathology, Institute of Integrative Biology, Swiss Federal Institute of Technology (ETH) Zurich, CH-8092 Zurich, Switzerland; Plant Pathology, Institute of Integrative Biology, Swiss Federal Institute of Technology (ETH) Zurich, CH-8092 Zurich, Switzerland; Experimental Biology Research Group, Institute of Biology, University of Neuchatel, CH-2000 Neuchatel, Switzerland; Johannes-Gutenberg-University Mainz, Institute of Molecular Physiology, Microbiology and Biotechnology, 55128 Mainz, Germany; Department of Fundamental Microbiology, University of Lausanne, CH-1015 Lausanne, Switzerland; Plant Pathology, Institute of Integrative Biology, Swiss Federal Institute of Technology (ETH) Zurich, CH-8092 Zurich, Switzerland

**Keywords:** Interspecies interactions, host-sharing, coinfection, coexistence, biological control, insecticidal pseudomonads, entomopathogenic nematodes, nematode mutualistically associated bacteria

## Abstract

A promising strategy to overcome limitations in biological control of insect pests is the combined application of entomopathogenic pseudomonads (EPPs) and nematodes (EPNs) associated with mutualistic bacteria (NABs). Yet, little is known about interspecies interactions such as competition, coexistence, or even cooperation between these entomopathogens when they infect the same insect host. We investigated the dynamics of bacteria–bacteria interactions between the EPP *Pseudomonas protegens* CHA0 and the NAB *Xenorhabdus bovienii* SM5 isolated from the EPN *Steinernema feltiae* RS5. Bacterial populations were assessed over time in experimental systems of increasing complexity. *In vitro*, SM5 was outcompeted when CHA0 reached a certain cell density, resulting in the collapse of the SM5 population. In contrast, both bacteria were able to coexist upon haemolymph-injection into *Galleria mellonella* larvae, as found for three further EPP-NAB combinations. Finally, both bacteria were administered by natural infection routes i.e. orally for CHA0 and nematode-vectored for SM5 resulting in the addition of RS5 to the system. This did not alter bacterial coexistence nor did the presence of the EPP affect nematode reproductive success or progeny virulence. CHA0 benefited from RS5, probably by exploiting access routes formed by the nematodes penetrating the larval gut epithelium. Our results indicate that EPPs are able to share an insect host with EPNs and their mutualistic bacteria without major negative effects on the reproduction of any of the three entomopathogens or the fitness of the nematodes. This suggests that their combination is a promising strategy for biological insect pest control.

## Graphical Abstract

**Graphical Abstract:**
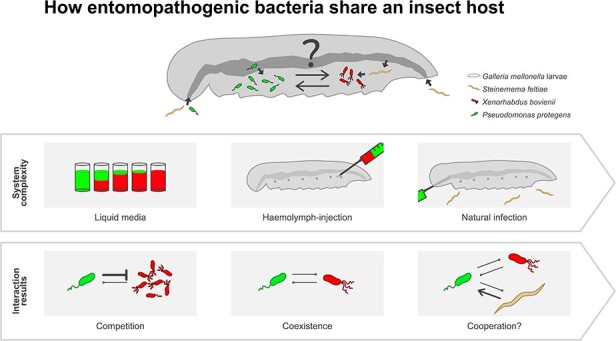

## Introduction

Insect pests contribute to severe yield losses in agriculture worldwide [1, 2] and are often fought with insecticides; a strategy, however, associated with environmental and human risks [3, 4]. Below-ground insect pests are particularly difficult to control due to the absence or banning of suitable pesticides [5, 6]. An environmentally friendly alternative to insecticides is biological control, i.e. the use of living organisms to control a disease or pest [7]. The use of biocontrol agents (BCAs) has great potential, but their unreliable performance in the field needs to be overcome [8]. One way to improve biocontrol performance is the combined application of several BCAs [9, 10]. Some studies have already shown an improved plant protection [11], increased killing speed, or insect mortality [12-15] with the use of BCA combinations. However, depending on the BCA combinations and application techniques used, negative effects on insect control were observed, which several authors attributed to negative interactions between the BCAs [10, 13, 16].

When different entomopathogenic BCAs are applied in the same field, it is likely that these organisms interact with each other in the environment (e.g. soil, roots, and leaves) or when infecting the same insect. Within the same host, pathogens can compete for nutrients and space directly by exploiting resources or producing toxins, or indirectly by modulating the host immune response [17-19]. Alternatively, pathogens may cooperate to harm their host more efficiently, share public goods, or cross-feed each other [20]. Understanding these ecological aspects is a prerequisite for successful application of BCA combinations. For example, if the reproduction of the BCAs in combined treatments is impaired, inoculative biocontrol may be severely compromised.

In this study, we investigated the interactions between entomopathogenic pseudomonads (EPPs) and entomopathogenic nematodes (EPNs) with their mutualistically associated bacteria (NABs), both long-studied and promising BCAs. In previous studies, the combination of these two BCAs showed increased protection of radish against the cabbage root fly in lab and semi-field experiments [11] as well as increased killing efficacy against two different insect species [12]. The EPPs *Pseudomonas protegens* and *Pseudomonas chlororaphis* are aggressive root colonizers with plant growth-promoting and disease-suppressing abilities [21-24]. In addition, these versatile bacteria colonize and infect insect larvae following oral uptake [25, 26]. Once ingested, they need to withstand the harsh conditions of the insect gut, compete with gut microbes, and cross the gut epithelial barrier [27, 28]. Once in the haemolymph they produce the insect toxin Fit [29], overcome the insect’s immune system, and proliferate rapidly to eventually kill the insect [28]. Their ability to thrive in different environments is partly due to their high competitiveness. EPPs produce a wide range of antimicrobials such as 2,4-diacetylphloroglucinol (DAPG), phenazines, pyoluteorin, or hydrogen cyanide [30]. Additionally, type VI secretion systems (T6SS) and tailocins contribute to the specific killing of bacteria [27, 31].

The likewise versatile NABs of the genera *Xenorhabdus* and *Photorhabdus* are mutualistically associated with EPNs of the genera *Steinernema* and *Heterorhabditis*, respectively [32]. Upon entering an insect, the nematodes release the NABs into the insect haemolymph where the bacteria multiply to high numbers. Both organisms produce several insect toxins leading to rapid host death [33, 34]. The nematodes reproduce in the carcass and, as nutrients become exhausted, differentiate into the free-living form called infective juveniles (IJs). IJs reassociate with their NABs and leave the carcass to infect a new host [32]. NABs not only kill the insect but also exclude other microorganisms from the carcass, mainly through the production of different antimicrobials such as stilbenes, carbapenem, indoles, xenorhabdins [32, 35], and compounds with highly specific antibacterial activity such as evybactin [36].

There is increasing evidence that EPN–NAB interactions are not monoxenic, but that specific bacterial species are often associated with EPNs [37]. The ecological relevance of these bacteria to EPNs is not yet clear. Among others, *Pseudomonas* spp. and sometimes even *P. protegens* and *P. chlororaphis* were detected on EPNs or in infected carcasses. They were initially thought to be contaminants, but more recent studies have shown that they are rather closely associated with EPNs [37]. Cambon *et al*. [38] detected *Pseudomonas* spp. with high frequency in nematode-infected carcasses at one of their sampling sites. *P. protegens* and *P. chlororaphis* were described as part of the EPN pathobiome by Ogier *et al*. [39] and Ruiu *et al*. [40] suggested a *P. protegens* strain to be closely associated with *Steinernema feltiae* as it was reisolated from IJs and infected carcasses over several rearing cycles. These studies suggest that EPPs and EPNs co-occur in nature, but for a successful biocontrol application it is important to know how EPPs interact with EPNs and their mutualistic NABs inside an insect. We have two hypotheses: firstly, EPNs and NABs compete or secondly, they coexist and, at best, interact synergistically. Here, we define coexistence as the ability of two organisms to colonize and multiply in the same host without substantially decreasing each other’s population sizes. The first hypothesis is supported by two studies describing the suppression of NABs by EPPs *in vitro* [39, 41]. Both bacteria can colonize and kill insect larvae and produce numerous antimicrobials; a competition for the same resource-rich space seems therefore likely. The second hypothesis is supported by the findings of co-occurrence and association of EPPs with EPNs in nature [38-40]. In addition, our previous work showed that a *P. chlororaphis* strain and a *Xenorhabdus bovienii* strain can co-colonize larvae of two insect species [12]. What is lacking is a detailed understanding of the interaction dynamics between EPPs and NABs inside insects and the effect of EPPs on EPN fitness. Here, we addressed these gaps by investigating the interaction between *P. protegens* CHA0 as a representative of EPPs and *X. bovienii* SM5 associated with the nematode *S. feltiae* RS5 as representatives of NABs and EPNs, respectively. Bacteria–bacteria interactions were studied in experimental systems of increasing complexity: first *in vitro*, then inside larvae of the greater wax moth *Galleria mellonella* that either excluded the nematodes or allowed infection via the natural route, i.e. orally for EPPs and EPN-vectored for NABs. In the latter, we also investigated the effects of EPPs on nematode fitness and the potential vectoring of EPPs by EPNs. We provide evidence that EPPs and EPNs can coexist *in vivo* and that EPPs even benefit from EPNs, legitimizing their combined application for biological insect control.

## Methods

### Organisms

Bacterial strains (Table 1) were grown overnight in lysogeny broth (LB) [42] at 24°C and adjusted to the desired concentrations by measuring optical density at 600 nm.

**Table 1:** Entomopathogens used in this study; the fluorescent tag information in the strain names has been omitted in the text for simplicity.

**Species**	**Strain**	**Characteristics**	**Reference**
*Pseudomonas protegens*	CHA0-gfp	CHA0::*att*Tn*7*-*gfp2*; Gm^R^	[29]
*Pseudomonas chlororaphis*	PCLRT03-gfp	PCLRT03:: *att*Tn*7*-*gfp2*; Gm^R^	[11]
*Xenorhabdus bovienii*	SM5-mche	SM5::16S-*mcherry*; Kan^R^	[12]
*Photorhabdus laumondii*	DJC-mche	DJC::*mcherry*; Km^R^	[67] tagged by group of Prof. Ralf Heermann, IMP, JGU Mainz
*Steinernema feltiae*	RS5-mche	Reassociated with SM5-mche	[12]

Nematodes (Table 1) were propagated in *G. mellonella* larvae using kanamycin (10 μg/larva) injections as a pretreatment as previously described [12]. IJs were stored at 15°C in tap water in tissue culture flasks and adjusted to the desired concentrations before use.

*G. mellonella* neonates were reared on pollen and later instars on a wood shaving-food mixture for 3–6 weeks at 24°C in the dark [43]. Last-instar larvae were stored at 10°C for up to 28 days. Larvae weighing between 0.16 and 0.22 g were used for *in vivo* assays and heavier larvae were additionally used for virulence assays of IJs.

Further details on the culturing and rearing of the organisms can be found in the Supplementary Methods.

### Experimental systems

**Figure 1:**
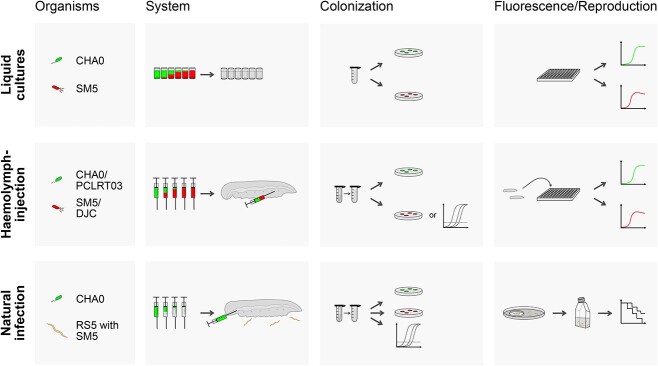
**Schematic drawing of the main experimental systems and analysis methods.** Different experimental systems were designed to observe interspecies interactions between entomopathogens, using three complexity levels. Entomopathogenic pseudomonads (EPPs) and nematode mutualistically associated bacteria (NABs) were incubated in LB as liquid cultures. Haemolymph-injection was used to observe interactions *in vivo* in larvae of *G. mellonella.* In the natural infection system, larvae were force-fed with EPPs and then exposed to entomopathogenic nematodes (EPNs) with NABs. This mimics more natural infection routes of the bacteria and integrates the effect of the nematodes into the system. In all systems, single entomopathogens and combinations with different inoculum ratios were used for inoculation or infection. Bacterial proliferation was monitored either by selective plating of cultures or homogenized larvae for the determination of colony forming units (CFUs) (Figs 2 and 6) or by measuring relative fluorescence units (RFUs) in different channels (Fig. 5) to discriminate differentially labeled bacteria (green, Gfp; red, mCherry). As *Photorhabdus laumondii* DJC cannot be reliably plated for CFU determination, qPCR was used to determine relative cell numbers instead of CFUs (Fig. 6). In the natural infection approach, the effects of *P. protegens* CHA0 on the nematodes themselves were investigated by 1) qPCR to determine the relative biomass of nematodes during reproduction, 2) determining emergence time point and the numbers of infective juveniles (IJs) leaving the carcass, and 3) testing the virulence of the emerged IJs (Fig. 3).

For experiments involving bacteria, cultures were adjusted to a final concentration of ~1 x 10^6^, 1 x 10^4^, or 1 x 10^2^ cells per 100 μl LB (*in vitro* assays) or per larva (*in vivo* assays) per strain. Sterile LB served as a negative control. For the bacterial combinations, strains were mixed at different ratios shortly before application. Experiments involving EPNs were performed with 80 or 160 IJs/larva and tap water served as a negative control. Experiments were incubated at 24°C and larvae were kept on filter paper in Petri dishes. Experimental systems are summarized in Fig. 1 and detailed descriptions of the experimental steps can be found in the Supplementary Methods.

For the *in vitro* assays, the adjusted single and combined bacterial cultures were incubated in LB in 96-well plates. One plate was incubated in a multimode microplate reader to measure fluorescence intensity in relative fluorescence units (RFUs) over time. The second plate was incubated to estimate colony-forming units (CFUs) by selective plating at 0, 5, 10, 24, and 48 hours post inoculation (hpi). The experiment was performed twice with six replicate wells per treatment.

Two different *in vivo* assays were performed with *G. mellonella* larvae. Haemolymph-injection: Larvae were injected with 10 μl bacterial suspension. Natural infection: Larvae were force-fed with 10 μl CHA0 suspension, then 80 IJs/larva were added in 400 (55-mm Petri dish) or 1000 μl (90-mm Petri dish) tap water. IJs were expected to contain 10–100 SM5 cells based on results obtained for similar *Steinernema* spp. [44, 45] resulting in ~800–8000 initial cells/larva in our experiments. Each experiment was performed twice with 22–31 larvae per treatment. The fluorescence emitted by eight larvae was measured using a multimode microplate reader, five to eight larvae were homogenized at 1, 3, and 6 dpi to determine bacterial colonization, and three to four larvae were observed under the fluorescence stereomicroscope from 1–9 dpi.

Bacterial colonization of the larvae was assessed by determination of CFUs following selective plating or relative cell numbers by quantitative polymerase chain reaction (qPCR). Larvae were homogenized in 1 ml LB as previously described [46] resulting in 1250 μl homogenate for an average larva. Alive larvae were additionally surface-disinfected before homogenization as previously described [12]. The homogenate was plated directly or frozen for later qPCR analysis.

Relative nematode biomass inside the larvae was measured in the natural infection assay using qPCR. Five larvae per treatment from two independent experiments were investigated. All following experiments were performed with IJs emerged from three independent repetitions of the natural infection assays. For IJ emergence and virulence, 12 dead larvae per treatment were placed in pairs on White traps [47]. Each individual carcass was regularly observed for newly emerging IJs, characterized as streams of IJs emerging from the carcass, and IJs were harvested and counted per white trap (i.e. IJs emerging from two larvae) from 8–21 dpi. Equal numbers of IJs from all White traps were pooled per treatment to determine virulence. They were surface-disinfected in 70% EtOH for 1 min and thoroughly rinsed with sterile tap water, rinsed only, or not treated at all. One day after surface-disinfection, 21 larvae were infected with 160 IJs/larva and survival was monitored for 51 h. No reaction to poking was rated as death. The effect of surface-disinfection itself and the effect of CHA0 during RS5 reproduction on IJ virulence were tested.

In the vectoring assays, we investigated the presence of CHA0 in and on the same IJs harvested for the virulence assay and in larvae reinfected with these IJs. For each treatment, 100 μl nematode sample containing 200 IJs in tap water was homogenized for 1 min using pestle and electric grinder. The pestle was washed with 100 μl LB and the homogenate was plated on selective medium. The remaining IJs were used to reinfect *G. mellonella* larvae, either by adding 160 IJs/larva directly to the larvae or behind a barrier. The latter was only done in one experimental repetition and achieved by pipetting IJs onto a filter paper in the lid of an inverted 30-mm Petri dish. This dish was closed and added to larvae on a filter paper within a 90-mm Petri dish. This avoided direct contact between larvae and nematodes, forcing the nematodes to crawl over the edge of the lid to search and infect the larvae. Colonization by CHA0 was determined by selective plating of five larval homogenates per treatment at 6 dpi.

### Statistics

Data were analyzed with R using R-studio (version 2023.06.0). Data from all independent experiments were pooled for plotting and statistical analysis. Colonization data (CFUs, cells, or IJs) and IJ emergence numbers were compared on log10-transformed data using a linear mixed effect model (package: lme4 [48]), followed by a post-hoc pairwise comparison of estimated marginal means (package: emmeans [49]) accounting for differences between experiments. Detection limits of CFUs varied depending on species, specific experiments, and time points. Samples below detection limits were set to ½ log of the lowest detection limit or 0. Survival and emergence curves were analyzed using Kaplan–Meier-based survival plots and treatments were compared using a Cox model followed by a post-hoc pairwise comparison (packages: coxme [50] and emmeans [49]) accounting for differences between experiments.

## Results

### *P. protegens* CHA0 outcompetes *X. bovienii* SM5 *in vitro*, but not *in vivo*, and benefits from *S. feltiae* RS5 infection

We investigated the dynamics and outcome of bacteria–bacteria interactions between *P. protegens* CHA0 and *X. bovienii* SM5 (isolated from *S. feltiae* RS5) in three different experimental systems: liquid cultures and *G. mellonella* larvae following either haemolymph-injections or natural infection.

**Figure 2:**
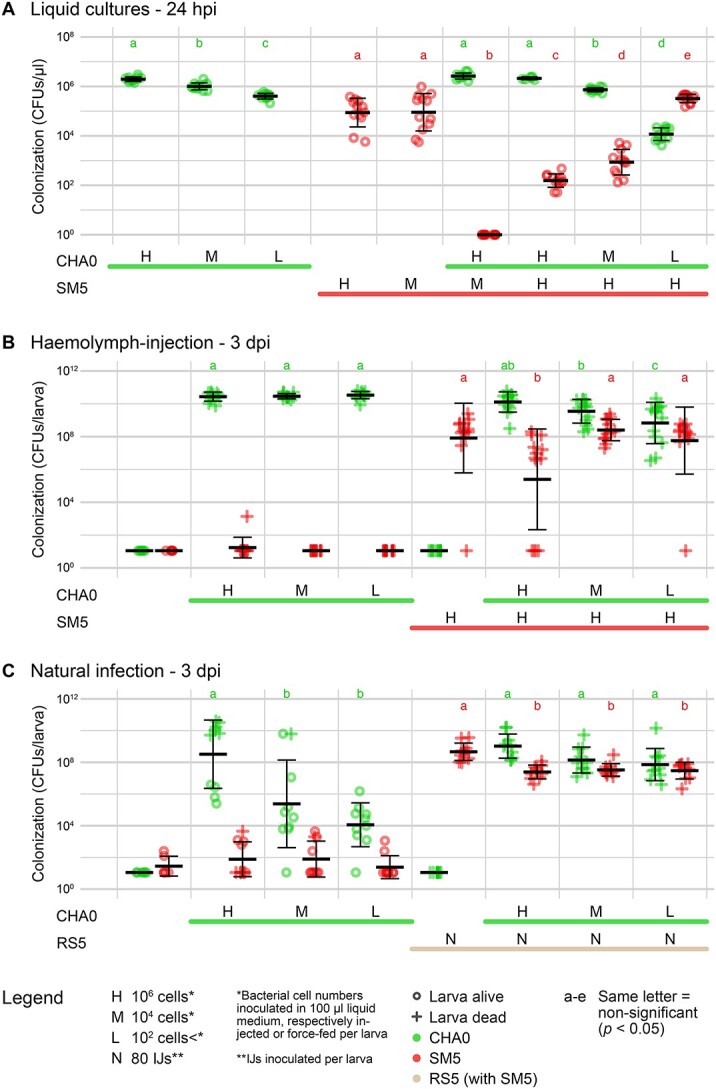
**Interactions between *P. protegens* CHA0 and *X. bovienii* SM5 *in vitro* and in *G. mellonella* larvae.** The proliferation of *P. protegens* CHA0 and *X. bovienii* SM5 was monitored in single and combined treatments after (**A**) inoculation in LB, (**B**) haemolymph-injection into *G. mellonella* larvae, or (**C**) natural infection of *G. mellonella* larvae with CHA0 and *S. feltiae* RS5 associated with SM5. Colony forming units (CFUs) per μl or per larva were determined by plating liquid cultures or homogenized larvae on selective media. Liquid cultures were analyzed at 0, 5, 10, 24, and 48 h post inoculation (hpi) and *G. mellonella* larvae at 1, 3, and 6 days post infection (dpi) (for all time points and individual experiment data see Figs S1–3). Individual data points are shown for CHA0 and SM5 with mean and standard deviation. Lines and capital letters below the graphs indicate which organisms and in which quantities (H = 10^6^ cells, M = 10^4^ cells, L = 10^2^ cells, *N* = 80 IJs) were added to the respective treatment. Each experiment was repeated once (*N* = 2), and data were pooled for plotting and statistical analysis. Lowercase letters (a-e) indicate significant differences between treatments according to a linear mixed effect model and post-hoc pairwise comparison and can be compared when written at the same height.

When inoculated alone in LB, CHA0 proliferated to 10^6^–10^7^ CFUs/μl and SM5 to 10^4^–10^6^ CFUs/μl (Figs 2A and S1). The proliferation of CHA0 was slightly impaired by SM5 when inoculated at a 1:10 000 (24 and 48 hpi) or 1:100 (24 hpi) cell ratio; however, CHA0 could still grow exponentially even when initially outnumbered 10 000-fold by SM5. At 10 hpi, SM5 cell numbers were elevated in most combinations indicating an accelerated proliferation due to the presence of CHA0. Yet, already at 24 hpi, SM5 was largely outnumbered by CHA0 in most combinations and at 48 hpi SM5 cells could no longer be detected. This indicates that after an initial proliferation of SM5, the presence of CHA0 led to the collapse of the SM5 population.

Bacteria were injected into the larval haemolymph (Figs 2B and S2), resulting in 10^9^–10^10^ CFUs/larva for CHA0 and 10^6^–10^9^ CFUs/larva for SM5 in single infections. As observed in the *in vitro* experiments, CHA0 was only slightly affected by the presence of SM5 when inoculated at a cell ratio of 1:100 or 1:10 000. Even if initially outnumbered 10 000-fold, CHA0 still managed to reach ~10^8^ CFUs/larva. SM5 was more competitive in the larval haemolymph than in the LB cultures. Only in the 1:1 ratio SM5 disappeared in most replicates at 6 dpi; in the other combinations it was not impacted.

In the natural infection assay, CHA0 single infections no longer resulted in lethal infections in all larvae (Figs 2C and S3). The more CHA0 cells were force-fed, the more larvae died. CHA0 grew to 10^9^–10^10^ CFUs/larva, indicating that it was able to cause systemic infection. In cases where CHA0 remained at 10^3^–10^6^ CFUs/larva, the bacteria probably did not reach the haemolymph. In the combinations, all larvae died and CHA0 was able to establish 10^7^–10^10^ CFUs/larva already at 3 dpi. Although CHA0 alone reached the highest population sizes, it caused systemic infection more consistently during EPN infection, suggesting that CHA0 was overall more successful in colonizing the larvae during EPN infection. SM5 proliferated to 10^7^–10^8^ CFUs/larva in all treatments and was only transiently affected by CHA0 at 3 dpi. A few cross-contaminations of SM5 in non-SM5 treatments occurred at low levels at 3 dpi, presumably during larval homogenization.

The interaction of the two bacterial species in the different experimental systems shifted from an outcompetition of SM5 by CHA0 to a coexistence and even indifference of SM5 toward CHA0. CHA0 was highly competitive in all systems, growing exponentially even when initially 10 000-fold outnumbered, and it appeared to benefit from the nematode infection.

### *P. protegens* CHA0 affects *S. feltiae* RS5 biomass inside larvae but not its progeny

Interactions between CHA0 and SM5 might alter the fitness of the nematode RS5, therefore different life-history traits of RS5 during and after the coinfection with CHA0 were investigated.

**Figure 3:**
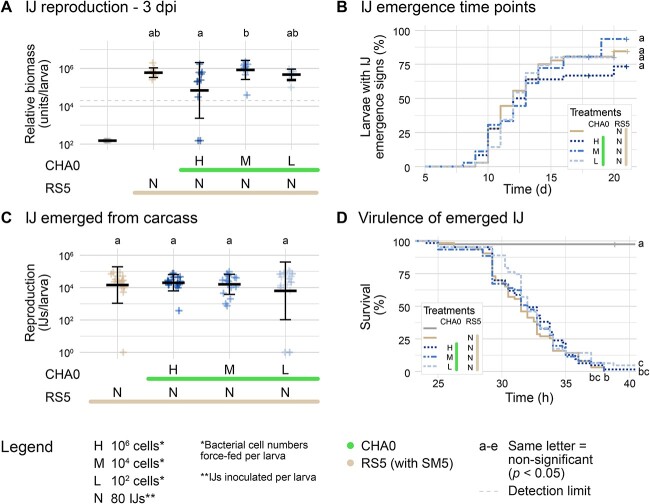
**Effect of *P. protegens* CHA0 on *S. feltiae* RS5 reproductive success and progeny virulence.** *G. mellonella* larvae were force-fed with *P. protegens* CHA0 or LB followed by infection with the nematode *S. feltiae* RS5 associated with *X. bovienii* SM5. The fitness of RS5 in presence of CHA0 was analyzed based on different life-history traits: Relative biomass of RS5 during reproduction in the larvae (**A**), time points (**B**) and numbers (**C**) of infective juveniles (IJs) emerging from the carcass, and the virulence of these IJs against *G. mellonella* larvae (**D**). Relative nematode biomass was determined by qPCR from homogenized larvae at 1, 3, and 6 dpi (for all time points and individual experiment data see Figs S4–S7). Dead larvae were transferred to White traps and regularly checked for signs of IJ emergence from 8–21 dpi. Emerging IJs were collected, counted, surface-disinfected, and tested for virulence. Lines and capital letters below the graphs or in the treatment box indicate which organisms and in which quantities (H = 10^6^ cells, M = 10^4^ cells, L = 10^2^ cells, *N* = 80 IJs) were added to the respective treatments. Relative nematode biomass and numbers of emerged IJs are shown per larva as individual data points with mean and standard deviation (A, C). Kaplan–Meier curves show the percentage of larvae with signs of IJ emergence (B) or larval survival after exposure to emerged IJs (D). Experiments were repeated once (A: *N* = 2) or twice (B–D: *N* = 3), and data were pooled for plotting and statistical analysis. Lowercase letters (a-e) refer to significant differences between treatments according to a linear mixed effect model (A, C) or a Cox model (B, D) and post-hoc pairwise comparisons.

The relative biomass of RS5 quantified by qPCR generally reached 10^6^–10^7^ units/larva without being impacted by CHA0 in any treatment except the highest CHA0 feeding concentration at 6 dpi (Figs 3A and S4). Thus, the reproduction within the larvae appeared to be affected toward the end following initial force-feeding of CHA0 at high concentrations. To verify this finding, nematode progeny produced in the presence or absence of CHA0 was examined by monitoring IJ emergence time point (Figs 3B and S5), numbers (Figs 3C and S6), and virulence (Figs 3D and S7). Emergence started at 8–9 dpi and new emergence events plateaued at 16 dpi. Although not significant, slightly less larvae showed emergence signs in the highest CHA0 feeding concentration after 15 dpi. In all treatments, 10^4^–10^5^ IJs/larva emerged by 21 dpi without any differences between single and combination treatments. The collected IJs were surface-disinfected and then tested for differences in killing speed in a *G. mellonella* virulence assay. 50% of the larvae were dead at around 32 hpi. The virulence of RS5 was not impacted by either CHA0 or the surface-disinfection.

Although RS5 biomass was affected by high CHA0 feeding concentrations, we could not detect any pronounced effect of CHA0 on the RS5 progeny as neither IJ emergence time point, numbers, nor virulence were statistically significantly altered. We conclude that the nematodes might be affected by high initial CHA0 concentrations but that this effect is no longer relevant when IJs are developing in the cadaver.

### *P. protegens* CHA0 is not vectored to new hosts by *S. feltiae* RS5

**Figure 4:**
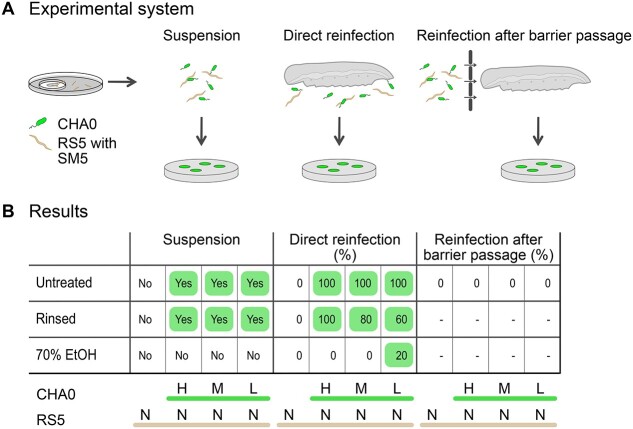
**Vectoring of *P. protegens* CHA0 by *S. feltiae* RS5 to new hosts.** Infective juveniles (IJs), harvested from *G. mellonella* larvae force-fed with different quantities (H = 10^6^ cells, M = 10^4^ cells, and L = 10^2^ cells) of *P. protegens* CHA0 followed by infection with the nematode *S. feltiae* RS5 associated with *X. bovienii* SM5 (*N* = 80 IJs), were tested for their ability to vector CHA0 to new hosts. The experimental systems are shown in **A**, the results in **B**. Emerging IJs were collected, exposed to different procedures (no treatment (untreated), rinsing with water (rinsed), surface-disinfection with 70% EtOH (70% EtOH)), and added to *G. mellonella* larvae for direct reinfection or in a system where the nematodes needed to pass a barrier to reach the larvae for reinfection. The presence of CHA0 in the IJ samples and the percentage of dead larvae colonized with CHA0 was determined by selective plating of homogenized IJs or larvae. Experiments were conducted three times (*N* = 3), except for reinfection after barrier passage, which was performed once. Data were pooled resulting in a total of three replicates of suspensions samples, 15 homogenized larvae for direct reinfection, and five homogenized larvae for reinfection after barrier passage per treatment.

The coexistence of RS5 and CHA0 in the larvae led us to hypothesize that CHA0 might be vectored by the IJs to the next host. To test this hypothesis, we performed different experiments (Fig. 4A) with the IJs harvested for the virulence assays before.

We homogenized part of the IJs directly from the harvested suspensions, after rinsing, or after surface-disinfection with 70% EtOH and used selective plating to determine the presence of CHA0. CHA0 was detected in all IJ samples, except in the surface-disinfected ones (Fig. 4B). These results suggest that CHA0 emerges with the IJs and is probably present on their surface but unlikely beneath the cuticle sheath. The second part of the IJs was used to reinfect larvae directly or after forcing them to overcome a physical barrier (Fig. 4B). Adding untreated or rinsed IJs directly to the larvae resulted in most cases in carcasses infected with CHA0. This occasionally occurred with surface-disinfected IJs in which we had not previously detected CHA0, suggesting that in rare cases CHA0 was present inside IJs. In the untreated or rinsed IJ samples, CHA0 was present in the suspensions added to the filter paper and we cannot exclude the possibility that larvae got infected with CHA0 by nibbling on the filter paper rather than via RS5 vectoring. To exclude this possibility, we separated the IJ suspension from the larvae with a barrier. There, none of the RS5-infected carcasses contained CHA0, indicating that RS5 most likely did not carry CHA0 over the barrier and into the larvae.

Overall, these results indicate that CHA0 left the carcass with RS5 but did not generally appear to be inside the IJs. We found no clear evidence that CHA0 used RS5 to reach new hosts.

### Fluorescence dynamics provide a rough indication on bacterial proliferation without destroying the insect

We tested if we could use the fluorescent tags carried by the bacteria (Table 1) to monitor their proliferation inside insects without destroying the body of the insect, similar to a previously described method [51]. Infected larvae were placed in 96-well plates and the fluorescence emitted by GFP-tagged CHA0 and mCherry-tagged SM5 was measured at regular time intervals. The RFUs were then compared with the CFUs obtained by selective plating performed in parallel.

**Figure 5:**
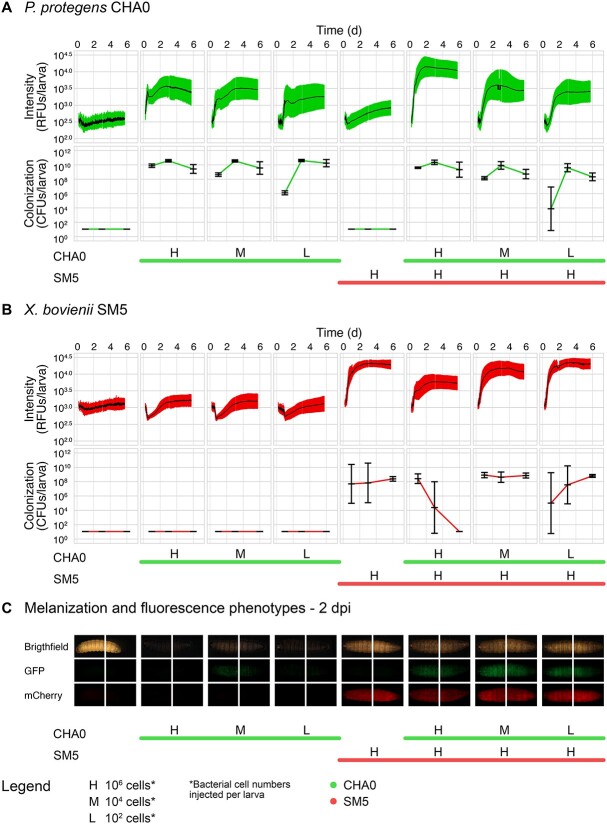
**Comparison of selective plating (colonization) and fluorescence detection (intensity), two methods to study the interactions between *P. protegens* CHA0 and *X. bovienii* SM5 in the haemolymph of *G. mellonella* larvae.** The proliferation of *P. protegens* CHA0 **(A)** and *X. bovienii* SM5 **(B)** was monitored in single and combined treatments after haemolymph-injection into *G. mellonella* larvae. In addition to selective plating, the fluorescence intensity emitted from GFP-tagged (CHA0) or mCherry-tagged (SM5) bacteria was measured through the larval body using a multimode microplate reader. This aimed at an indirect detection of the bacterial proliferation dynamics without destroying the insect. Relative fluorescence units (RFUs) per larva were measured over time using filters for GFP or mCherry detection. Means and standard deviations for CHA0 and SM5 are shown. Colony forming units (CFUs) per larva were determined by plating homogenized larvae on selective media at 1, 3, and 6 days post infection (dpi). Mean and standard deviation are shown. Images **(C)** show representative larvae (front and rear) at 2 dpi under brightfield (showing melanization levels), GFP, or mCherry conditions. Lines and capital letters below the graphs indicate which organisms and in which quantities (H = 10^6^ cells, M = 10^4^ cells, and L = 10^2^ cells) were added to the respective treatments. The experiment was conducted twice (*N* = 2). The repetition (experiment 2) is shown in Fig. S8 and comparisons of CFUs/larva with RFUs/larva under *in vitro* conditions are shown in Figs S9 and S10.

In the beginning, both fluorescence signals were highly variable, although at low levels, probably due to larval movements (Figs 5A and B, S8A and B). A significant increase in the RFUs of the expected signal was measured in all treatments, whereas no such increase was observed in treatments lacking the respective bacterial species. The presence of tagged bacteria could therefore be reliably measured through the intact larval body. The GFP signal roughly followed the increase in CHA0 CFUs; however, there was a brief quenching of the GFP signal in the single injections, but not in the combinations, shortly after the signal started to increase exponentially. The mCherry signal did not reflect the SM5 CFU dynamics well. The suppression of SM5 by CHA0 at the 1:1 inoculum ratio was visible, but the mCherry signal was still quite intense even though the CFU levels dropped to zero at 6 dpi.

Melanization, which occurs as part of the insect’s immune response [52], was monitored in parallel taking images of larvae (Figs 5C and S8C). Melanization was strongly induced in CHA0 single injections, where larvae turned black, but was suppressed by SM5 alone as larvae remained brownish. In the combinations, melanization was found at intermediate levels between the single treatments. This indicates that melanization is actively suppressed by SM5 even in the presence of CHA0.

We observed roughly similar dynamics between CFUs and RFUs in liquid cultures (Figs S9 and 10). In summary, the fluorescence signal gives a rough indication of bacterial proliferation, although melanization quenches the signal making it difficult to infer bacterial colonization in intact larval bodies from the RFU data.

### Different species of EPPs and NABs coexist *in vivo*

So far, we have only focused on CHA0 and SM5, yet we were interested to see whether our findings also apply to other EPP–NAB interactions after haemolymph-injection. We therefore tested *Photorhabdus laumondii* DJC as an additional NAB against CHA0, and the EPP *P. chlororaphis* PCLRT03 against SM5 and DJC.

**Figure 6:**
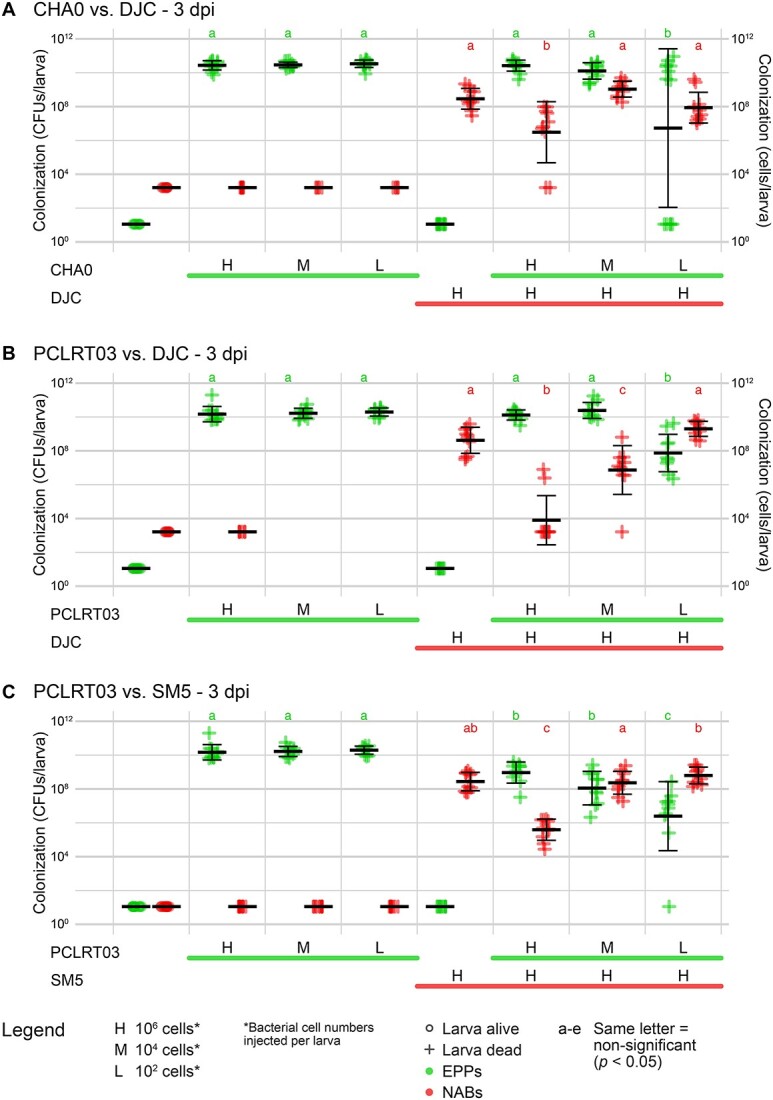
**Interactions between different entomopathogenic pseudomonads and nematode-associated bacteria in *G. mellonella* larvae following haemolymph-injection.** The proliferation of entomopathogenic pseudomonads (EPPs) (*P. protegens* CHA0, *P. chlororaphis* PCLRT03) and nematode mutualistically associated bacteria (NABs) (*X. bovienii* SM5, *P. laumondii* DJC) was monitored in single and combined treatments after haemolymph-injection into *G. mellonella* larvae. Interactions between CHA0 and DJC (**A**), PCLRT03 and DJC (**B**), or PCLRT03 and SM5 (**C**) were monitored. Bacteria were quantified in larval homogenates by selective plating (colony forming units (CFUs)/larva; CHA0, PCLART03, SM5) or by estimating relative cell numbers (cells/larva; DJC) using qPCR. Bacteria were monitored at 3 and 6 days post infection (dpi) and additionally at 1 dpi for CHA0 vs. DJC (for all time points and individual experiment data see Figs S11–S13). Individual data points are shown for EPPs and NABs with mean and standard deviation. Lines and capital letters below the graphs indicate which organisms and in which quantities (H = 10^6^ cells, M = 10^4^ cells, and L = 10^2^ cells) were added to the respective treatments. Each experiment was conducted twice (*N* = 2), and data were pooled for plotting and statistical analysis. Lowercase letters (a-e) indicate significant differences between treatments according to a linear mixed effect model and post-hoc pairwise comparison and can be compared when written at the same height.

CHA0 proliferated to 10^9^–10^10^ CFUs/larva when injected alone and was affected by the presence of DJC at 3 and 6 dpi in one repetition when injected at a ratio of 1:10 000 (Figs 6A and S11). It is possible that fewer than the targeted 100 CFUs of CHA0 were initially injected in this repetition, making it more difficult for CHA0 to establish. Nonetheless, the results indicate an overall insensitivity of CHA0 to the presence of DJC. DJC proliferated to 10^8^–10^9^ cells/larva in single injections and, similar to SM5 (Figs 2B and S2), was negatively affected by the presence of CHA0 when injected at a 1:1 ratio. Nevertheless, coexistence of the two species was possible in most larvae.

PCLRT03 was able to grow to 10^9^–10^10^ CFUs/larva and DJC to 10^8^–10^9^ cells/larva (Figs 6B and S12). Both bacteria were impaired by the antagonist, PCLRT03 at the 1:10 000 ratio and DJC at the 1:100 and 1:1 ratios, but still able to coexist. SM5 proliferated to 10^9^–10^10^ CFUs/larva in single injections and had a negative effect on all combinations with PCLRT03, which conversely only affected SM5 at the 1:1 ratio (Figs 6C and S13). In all combinations at 6 dpi, one of the two strains was affected by the other (CFUs often below the detection limit), indicating that the bacteria were less able to coexist than in any of the other combinations tested.

In most combinations, EPPs and NABs were able to coexist at least until 3 dpi. However, we found evidence that *P. laumondii* DJC was more affected by EPPs than *X. bovienii* SM5. This is shown by the trend that DJC was more often negatively affected in its proliferation by the EPPs and less often had a negative effect on them. Compared with *P. protegens* CHA0, *P. chlororaphis* PCLRT03 seemed to be more sensitive to SM5 and similarly insensitive to DJC.

## Discussion

In this study, we tested two hypotheses regarding the interaction dynamics between bacterial strains belonging to EPPs or NABs, and their impact on EPNs. As both bacteria can inhabit the same environment and are known to produce antimicrobials [30, 32, 35], a certain degree of competition was expected. Yet, coexistence seems plausible to some extent as *Pseudomonas* spp. are often found in EPN-infected carcasses [38-40].

In the first part of this study, the proliferation of *P. protegens* CHA0 and *X. bovienii* SM5 was monitored in single and combination treatments in experimental systems of increasing complexity. In combined liquid cultures, both bacteria initially proliferated, but SM5 was eventually eliminated. This is consistent with two studies in which EPPs were able to outcompete NABs *in vitro* [39, 41]. In contrast to the *in vitro* system, neither CHA0 nor SM5 outcompeted the other in any of the *in vivo* systems. Both were able to proliferate and coexist. We speculate that coexistence between EPPs and NABs *in vivo* might be a generalizable trait, as we were able to demonstrate it for three further NAB–EPP interactions involving different bacterial species. This rejects the hypothesis of a strong bacterial competition and confirms our previous study where EPPs and NABs could coexist in larvae of two agriculturally relevant pest insects [12]. The discrepancy between the experimental systems reflects the discrepancy found in literature and highlights that *in vitro* and *in vivo* results are not necessarily comparable. Few studies have investigated the interactions of either EPPs or NABs with other bacteria in insects, and all show genus-specific interactions [27, 53-55]. Negative interactions were often connected with the susceptibility of the antagonist to an antimicrobial of the NAB or EPP strain [27, 53-55].

Broad-spectrum antimicrobials may account for the outcompetition of SM5 by CHA0 *in vitro*, although cell contact-dependent killing mechanisms such as the T6SS may contribute as well. Vacheron *et al*. [27] showed that the T6SS is involved in the competition between CHA0 and commensal gut bacteria during larval infection. Interestingly, the decline of SM5 started when CHA0 reached the plateau phase. The production of certain antimicrobials such as DAPG and pyoluteorin is autoinduced and cell density-dependent in CHA0 [56, 57] and may play a role here. SM5 was less negatively affected by CHA0 as the system became more complex; this might be due to differential gene expression in CHA0 as well as in SM5. Vesga *et al*. [28] showed that most genes for the production of antimicrobials such as DAPG, pyoluteorin, pyrrolnitrin, or hydrogen cyanide were down-regulated in CHA0 when injected into the haemolymph of *G. mellonella* compared with growth in LB. This suggests that CHA0 does not produce high quantities of antimicrobials in the haemolymph, where the two bacteria most likely encounter each other, and may be the reason why SM5 is not eliminated in the *in vivo* systems. However, we cannot exclude that the presence of a competitor would induce the production of these antibiotics in the haemolymph. Another possible explanation for SM5 being less affected by CHA0 *in vivo* might be that SM5 upregulates resistance genes such as those encoding for efflux pumps in the haemolymph, thereby escaping the antimicrobials of CHA0. During the infection, NABs are thought to change their transcriptional profile including changes in genes responsible for the cell surface structure [58]. This might not only enable them to persist against the insect’s immune system, but additionally increase resistance to antimicrobials in general.

NABs are known to produce numerous antimicrobials [32, 35]. Yet, CHA0 appears to be unaffected by them in this study, allowing it to grow exponentially even when initially outnumbered 10 000-fold. This competitive proliferation bears similarities to the colonization of plant roots by EPPs. Competitive root colonization is one of the factors making CHA0 a promising BCA against soil-borne diseases and is thought to be based on the production of multiple antimicrobials, among other traits [59]. However, resistance to antimicrobials may also be important. Many members of the *Pseudomonas fluorescens* complex, including *P. protegens*, are resistant to multiple antibiotics and harbor several antimicrobial resistance genes in their genomes [60, 61]. Furthermore, the O-antigen polysaccharides of the outer membrane are involved in the resistance of CHA0 to cationic antimicrobial peptides [62] and tailocins [63], and are likely providing resistance to some of the NABs’ antimicrobials. For both EPPs and NABs, we cannot exclude the possibility that antimicrobials produced do not reach the antagonist due to compartmentalization within the carcass, and coexistence might be based on evasion rather than on antibiotic resistance. The small decreases in CFUs observed for CHA0 and SM5 at later time points *in vivo* are probably due to depleting nutrient sources in the decaying carcasses. Further clarification of the interaction mechanisms could be achieved by combining population monitoring with transcriptomics or proteomics at different stages of co-colonization.

The host of SM5, *S. feltiae* RS5, did not appear to be affected in coinfections except for a reduced relative biomass at 6 dpi when larvae were force-fed with high initial CHA0 concentrations. For lower concentrations, or when looking at IJ emergence numbers or virulence, CHA0 had no effect on RS5 progeny. This contradicts the finding that EPNs reproducing in competition with other entomopathogens or saprophytes often have reduced fitness [64, 65]. However, *Pseudomonas* spp. may be associated with EPNs as part of the second bacterial circle [37]. The basis for such an association would be that the interacting partners have no negative effect on each other. Our data suggest that the interaction is mostly neutral toward EPNs but positive toward EPPs. We hypothesize that CHA0 failed to cross the intestinal barrier when low inoculum concentrations were force-fed to *G. mellonella* larvae. Systemic infection sometimes occurred at higher inoculum concentrations, characterized by an exponential increase in CFUs, most probably in the haemolymph of the insect, leading to its death [46]. However, in combination with the nematode, CHA0 seemed to benefit from RS5 as it was able to proliferate to high cell numbers in all cases. In another study, commensal gut bacteria translocated into the insect haemolymph during gut epithelium penetration by an EPN and one bacterial species even proliferated besides the NAB [53]. It would be interesting to test with sterile nematodes whether CHA0 indeed benefits from the access routes provided by RS5 or rather from SM5 killing the insect. In either case, CHA0 and EPPs in general may be opportunistic pathogens in nature, able to persist in a healthy host and taking advantage of a weakened host, as previously suggested [46, 66]. Taken together, our results indicate that EPPs, EPNs, and NABs can coexist in an insect host in the sense of all three organisms being able to colonize and multiply inside the host without substantially decreasing each other’s population sizes.

In plants, CHA0 was shown to be vectored by flies to new hosts [46], thus we wondered whether the bacterium could also be vectored by EPNs. We found evidence for CHA0 leaving the carcass together with IJs, but only in rare cases for vectoring to new larvae, suggesting that this is unlikely to be a relevant mechanism of dispersal in natural systems. Coevolution of EPNs and EPPs could lead to more intimate relationships over time, as suggested for *P. protegens* CO1 isolated from *S. feltiae* [40]. Our *in vivo* systems would be ideal for future studies comparing EPPs isolated from EPNs with EPPs of other origins for coexistence, cooperation with, and vectoring by EPNs. To further develop the systems, we tested the use of fluorescence as a simple method to monitor bacterial interactions in a non-destructive way. The fluorescence signals gave a first impression of how the bacteria proliferated and competed, especially in the early stages of infection. However, it was not possible to study the interactions in detail due to the quenching effect of melanin.

Although we are just at the beginning of understanding the ecology of EPP-, EPN-, and NAB-interactions, our findings are highly relevant for the application of EPP–EPN combinations in biological insect control. The proliferation and coexistence of all three entomopathogens within the same host, as well as the conserved IJ virulence, indicates that EPNs and EPPs can be successfully combined. This is particularly important for inoculative biocontrol, which relies on the propagation of BCAs in the field.

## Supplementary Material

20240214_Supplementary_Information_wrae028

## Data Availability

All raw data analyzed during this study can be found on the research collection platform of ETH Zurich (doi: 10.3929/ethz-b-000652767).
