# Supplementary material for: Entomopathogenic pseudomonads can share an insect host with entomopathogenic nematodes and their mutualistic bacteria
Source: ISME J. 2024 Feb 21;18(1):wrae028. doi: 10.1093/ismejo/wrae028 (PMC10945363; doi:10.1093/ismejo/wrae028)
Supplement: 20240214_Supplementary_Information_wrae028 [file 20240214_supplementary_information_wrae028.pdf]

Supplementary Information

Supplementary Methods

Bacterial culturing
Galleria mellonella rearing
Haemolymph-injection
Force-feeding of larvae
Selective plating
qPCR
Fluorescence measurements
Microscopy of infected larvae

Supplementary Figures

Fig. S1: Interactions of *P. protegens* CHA0 and *X. bovienii* SM5 in vitro.
Fig. S2: Interactions between *P. protegens* CHA0 and *X. bovienii* SM5 in *G. mellonella* larvae following haemolymph-injection.
Fig. S3: Interactions between *P. protegens* CHA0 and *X. bovienii* SM5 in *G. mellonella* larvae following natural infection.
Fig. S4: *S. feltiae* RS5 reproductive success in *G. mellonella* larvae following natural infection.
Fig. S5: Infective juvenile (IJ) emergence time points for *S. feltiae* RS5 following natural infection.
Fig. S6: Infective juvenile (IJ) emergence numbers of *S. feltiae* RS5 following natural infection.
Fig. S7: Virulence of emerged infective juveniles (IJs) of *S. feltiae* RS5 following natural infection.
Fig. S8: Comparison of selective plating (colonization) and fluorescence detection (intensity), two methods to study the interactions between *P. protegens* CHA0 and *X. bovienii* SM5 in the haemolymph of *G. mellonella* larvae: Experiment 2.
Fig. S9: Comparison of selective plating (colonization) and fluorescence detection (intensity), two methods to study the interactions between *P. protegens* CHA0 and *X. bovienii* SM5 in vitro: Experiment 1.
Fig. S10: Comparison of selective plating (colonization) and fluorescence detection (intensity), two methods to study the interactions between *P. protegens* CHA0 and *X. bovienii* SM5 in vitro: Experiment 2.
Fig. S11: Interactions between *P. protegens* CHA0 and *P. laumondii* DJC in *G. mellonella* larvae following haemolymph-injection.
Fig. S12: Interactions between *P. chlororaphis* PCLRT03 and *P. laumondii* DJC in *G. mellonella* larvae following haemolymph-injection.
Fig. S13: Interactions between *P. chlororaphis* PCLRT03 and *X. bovienii* SM5 in *G. mellonella* larvae following haemolymph-injection.

References

Supplementary Methods

Bacterial culturing
Bacterial strains were stored in 40% (v/v) glycerol at -80°C and regrown at 24°C on King's B agar [1] supplemented with 13 µg/ml chloramphenicol, 100 µg/ml cycloheximide, and 10 µg/ml gentamycin (KB^{++G}) (EPPs) or on lysogeny broth (LB) agar [2] supplemented with 50 µg/ml kanamycin (LB^{K}) (NABs). For experiments, bacteria were cultured overnight in 20 ml LB with agitation (180 rpm) using sterile 100 ml shake flasks. Cultures were adjusted to the desired concentrations with sterile LB using optical density at 600 nm (OD_{600}) without prior washing steps. An OD_{600} of 0.2 corresponds to approximately 1 x 10^8 CFUs or cells/ml verified for all bacteria used here. EPPs were always stored on ice, whereas NABs were neither placed on ice nor vortexed to prevent cell death.

***Galleria mellonella* rearing**
G. mellonella were reared at 24°C in the dark, similar to the procedure described by Vicente-Diez et al. [3]. The moths were kept in pint-sized bug dorms for mating and oviposition. Eggs were collected on a filter paper placed on the nylon mesh covering the bug dorm and regularly transferred to pollen. Neonates were reared on pollen for 2-3 weeks and later instars on a wood shaving-food mixture in ventilated plastic containers for an additional 3-6 weeks. The food mixture contained 466 g dry homogenized dog food (Bitsdog, Landi, Switzerland), 266 ml of boiling tap water, 134 g honey (Aldi, Zurich, Switzerland), and 134 g 99.5% glycerine (FloraCura, London, UK) mixed with wood shavings to reduce the dampness and provide a substrate for the larvae to hide in. Last instar larvae were regularly harvested and stored at 10°C in well ventilated boxes containing wood shavings. Larvae used for rearing were incubated for 1-2 weeks at 24°C for pupation and subsequent moth hatching.

Haemolymph-injection
G. mellonella larvae (last instar, 0.18-0.22 g) were injected with 10 µl of the respective bacterial suspension or LB using insulin syringes (1 ml, medical; CODAN, Rødby, Denmark) with a sharp needle (27 G x ½''; Braun, Melsungen, Germany) attached to a DYMAX stepper (DYMAX, Torrington, CT, USA) similar to the procedure described by Vesga et al. [4]. Larvae were fixed between thumb and index finger. The needle was inserted at the sideline of the larvae behind the 4th pseudopods parallel to the larval body with the needle pointing towards the head. The larvae were released from the fingers and the 10 µl bacterial suspension was injected. Extensively bleeding larvae were discarded. The injected larvae were placed in batches of 5-10 in Petri dishes on filter paper and incubated at 24°C. Two to three injection rounds were made per treatment using freshly prepared syringes and needles.

Force-feeding of larvae
Trypan blue (0.4% (w/v)) was mixed 1:3 with the inoculum to verify successful delivery of the treatment to the larval gut. *G. mellonella* larvae (last instar, 0.18-0.22 g) were force-fed with 10 µl of the respective treatment using insulin syringes with a blunt needle (30 G x ½''; H. Sigrist & Partner, Matzingen, Switzerland) attached to a DYMAX stepper. For feeding, the larvae were fixed on their back with two fingers. The blunt needle was carefully inserted between the mandibles of the larvae for a minimum of 3 mm and 10 µl bacterial suspension was released while the pressure on the larvae from the finger was reduced. The needle was kept inside the larvae for approximately 2 s and then slowly removed. Larvae where more than 2-3 µl liquid exuded from the mandibles or which had turned blue due to internal injuries were discarded. The force-fed larvae were placed in Petri dishes on filter paper and incubated at 24°C. Two to three injection rounds were made per treatment using freshly prepared syringes and needles.

Selective plating

Liquid cultures or the larval homogenates were 10-fold serially diluted in 0.9% NaCl. The respective dilutions were spotted on LB^K selecting for SM5 and/or KB^{++G} selecting for CHA0 and PCLRT03. To determine cross-contamination, undiluted cultures or homogenates were spotted on the respective media. In the vectoring assay, the presence of CHA0 in the IJs or in larval homogenates was determined by plating 100 µl of the undiluted homogenate on KB^{++G} round plates using a Drigalski spatula. KB^{++G} and LB^K plates were incubated at 24°C until colonies became visible (2-3 days) and then stored at 3°C for CFU counting. Colony identity was verified using a fluorescence stereomicroscope (see "Microscopy of infected larvae"). Green fluorescence was used as evidence for GFP produced by CHA0 or PCLRT03 and red fluorescence for mCherry produced by SM5. Samples below the detection limit (in vivo experiments: 125 CFUs/larva for single treatments and 1250 CFUs/larva for combination treatment; all other assays: 0 CFUs/µl or 0 CFUs/larva) were set to ½ log of the lowest detection limit corresponding to 11.2 CFUs/larva in the in vivo assays and to 0 CFUs/µl or 0 CFUs/larva in all other assays.

qPCR

DNA was extracted from insect homogenates as described in Spescha et al. [5] with the following adjustments: starting material was collected from 114 µl homogenate. For this, the tissue was pelleted by centrifugation at 11'000x g for 3 min and the supernatant was discarded. Samples were lysed during 15-17 h and DNA was eluted with 200 µl elution buffer. qPCR for RS5 and DJC was performed as described by Spescha et al. [5] using the primer pair RpoDqPCR_fwDJC/RpoDqPCR_rvDJC for DJC [6]. DNA for the DJC standards was extracted from pure cultures corresponding to 3.7 x 10^8 cells of DJC as counted in a Thoma chamber using a fluorescence microscope. Cp values were correlated with DJC cell or RS5 IJ counts using a linear regression of the standard curve for each plate and thereby accounting for differences between qPCR runs. Relative DJC colonization (cells/larva) and relative biomass (units/larva) were calculated applying a cut-off of 26 or 24 Cp corresponding to a detection limit of 2.6 x 10^6 cells/larva or 2.3 x 10^4 units/larva, respectively. Samples below the detection limit were set to ½ log of the detection limit corresponding to 1.6 x 10^3 cells/larva and 1.5 x 10^2 units/larva, respectively.

Fluorescence measurements

The fluorescence intensity emitted by the tagged bacteria in cultures or in *G. mellonella* larvae was quantified in black 96-well plates (flat, clear bottom; Greiner Bio-One, Kremsmünster, Austria) using the Spark 10M multimode microplate reader (Tecan, Männedorf, Switzerland). The plates were incubated at 24°C with the supplied lids; bacterial cultures in liquid media were additionally sealed with parafilm. The fluorescence intensity of GFP (Ex: 485/20 nm, Em: 535/25) and mCherry (Ex: 580/20 nm, Em: 635/35), as well as of OD_{600} in liquid cultures, was measured every 15 min up to 6 dpi. Measurements in the liquid cultures were made after shaking for 5 s at 240 rpm. Liquid cultures were measured at one spot in the center of the well, larvae were measured at five spots per well and the mean of these five measurements was used for further calculations.

Microscopy of infected larvae

Larvae injected with SM5 and CHA0 were regularly observed using a fluorescence stereomicroscope Leica M205 FCA (Leica, Wetzlar, Germany) and images were taken to observe visual disease symptoms of larvae and spatial localization patterns of the fluorescence-labelled bacteria as described by Spescha et al. [5]. Dead larvae were observed directly, whereas live larvae were frozen for 20-40 min at -20°C to kill them. Image series under brightfield, GFP, and mCherry conditions were taken at 0.78x zoom of different parts of the larvae with 10, 400, and 400 ms exposure and 7, 6, and 8x gain, respectively. Lamp intensity was set to 30% for brightfield and switched off for fluorescence conditions. Images were not further processed.

Supplementary Figures

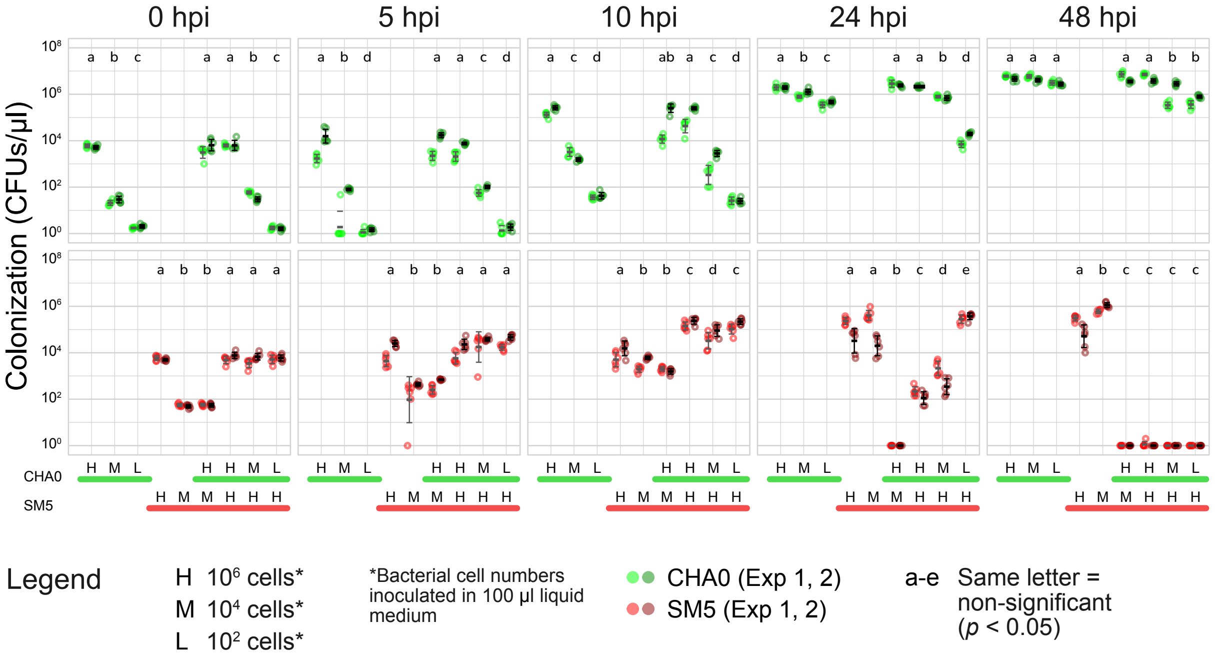

Fig. S1: **Interactions of *P. protegens* CHA0 and *X. bovienii* SM5 in vitro.**
The proliferation of *P. protegens* CHA0 and *X. bovienii* SM5 was monitored in single and combined treatments after inoculation in LB. Colony forming units (CFUs) per µl were determined by plating liquid cultures on selective media. Individual data points are shown in green for CHA0 and red for SM5 with mean and standard deviation. Coloured lines and capital letters below the graphs indicate which organisms (CHA0 = green, SM5 = red) and in which quantities (H = 10^6 cells, M = 10^4 cells, and L = 10^2 cells) were added to the respective treatment. Two replicate experiments (N=2) are shown, and data were pooled for statistical analysis. Lowercase letters (a-e) refer to significant differences between treatments according to a linear mixed effect model and post-hoc pairwise comparison.

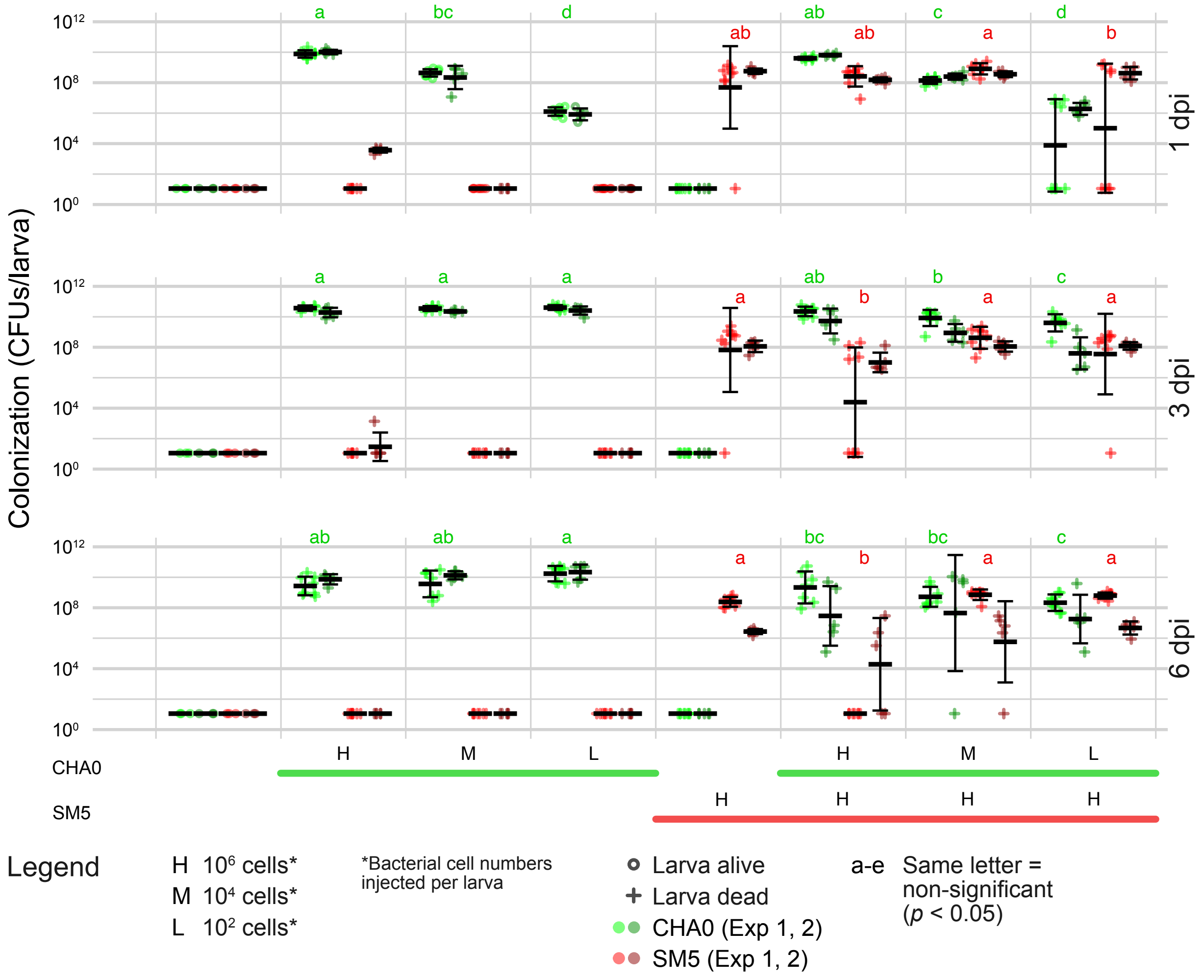

Fig. S2: **Interactions between *P. protegens* CHA0 and *X. bovienii* SM5 in *G. mellonella* larvae following haemolymph-injection**.

The proliferation of *P. protegens* CHA0 and *X. bovienii* SM5 was monitored in single and combined treatments after haemolymph-injection into *G. mellonella* larvae. Colony forming units (CFUs) per larva were determined by plating homogenized larvae on selective media. Individual data points are shown in green for CHA0 and red for SM5 with mean and standard deviation. Coloured lines and capital letters below the graphs indicate which organisms (CHA0 = green, SM5 = red) and in which quantities (H = 10^6 cells, M = 10^4 cells, and L = 10^2 cells) were added to the respective treatment. Two replicate experiments (N=2) are shown, and data were pooled for statistical analysis. Lowercase letters (a-e) refer to significant differences between treatments according to a linear mixed effect model and post-hoc pairwise comparison and can be compared when written in the same colour.

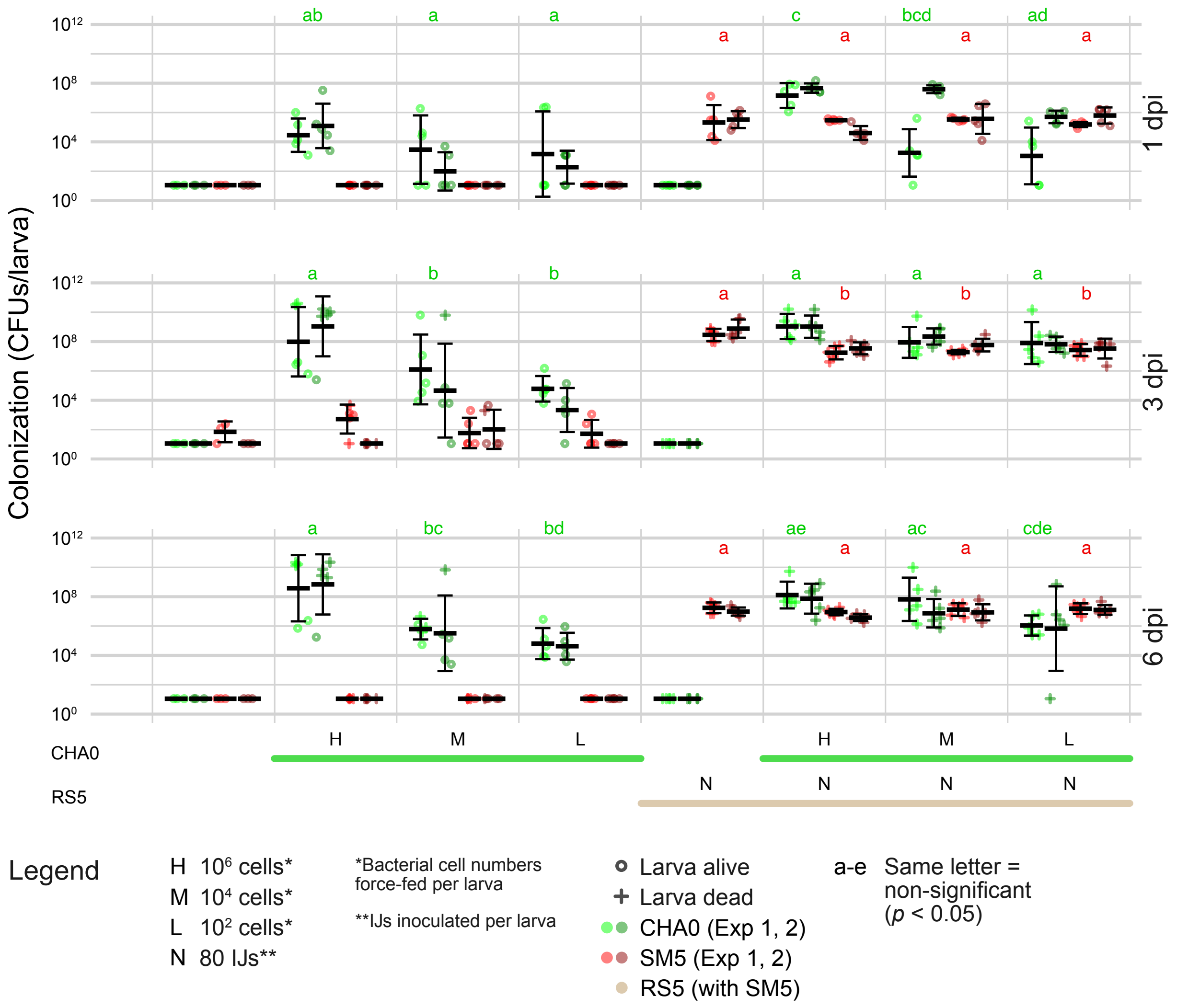

Fig. S3: **Interactions between *P. protegens* CHA0 and *X. bovienii* SM5 in *G. mellonella* larvae following natural infection.**

The proliferation of *P. protegens* CHA0 and *X. bovienii* SM5 was monitored in single and combined treatments after natural infection of *G. mellonella* larvae with CHA0 and *S. feltiae* RS5 associated with SM5. Colony forming units (CFUs) per larva were determined by plating homogenized larvae on selective media. Individual data points are shown in green for CHA0 and red for SM5 with mean and standard deviation. Coloured lines and capital letters below the graphs indicate which organisms (CHA0 = green, RS5 with SM5 = beige) and in which quantities (H = 10^6 cells, M = 10^4 cells, L = 10^2 cells, N = 80 Infective Juveniles (IJs)) were added to the respective treatment. Two replicate experiments (N=2) are shown, and data were pooled for statistical analysis. Lowercase letters (a-e) refer to significant differences between treatments according to a linear mixed effect model and post-hoc pairwise comparison and can be compared when written in the same colour.

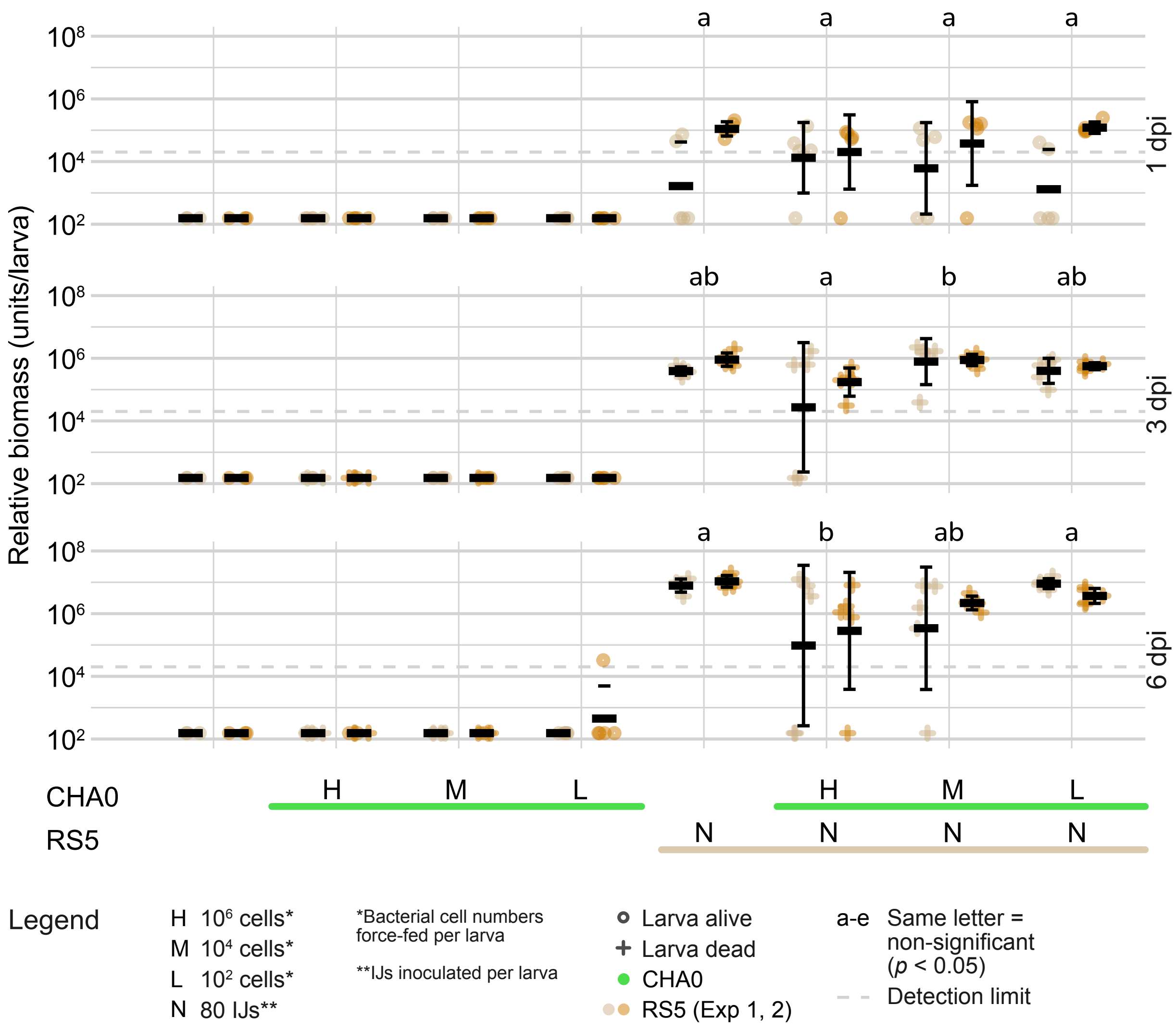

Fig. S4: ***S. feltiae* RS5 reproductive success in *G. mellonella* larvae following natural infection.**
G. mellonella larvae were force-fed with *P. protegens* CHA0 or LB followed by infection with the nematode *S. feltiae* RS5 associated with *X. bovienii* SM5. Relative nematode biomass was determined by qPCR from homogenized larvae. Coloured lines and capital letters below the graphs indicate which organisms (CHA0 = green, RS5 with SM5 = beige) and in which quantities (H = 10^6 cells, M = 10^4 cells, L = 10^2 cells, N = 80 infective juveniles (IJs)) were added to the respective treatments. Relative nematode biomass per larva is shown as individual data points with mean and standard deviation. Two replicate experiments (N=2) are shown, and data were pooled for statistical analysis. Lowercase letters (a-e) refer to significant differences between treatments according to a linear mixed effect model and post-hoc pairwise comparisons.

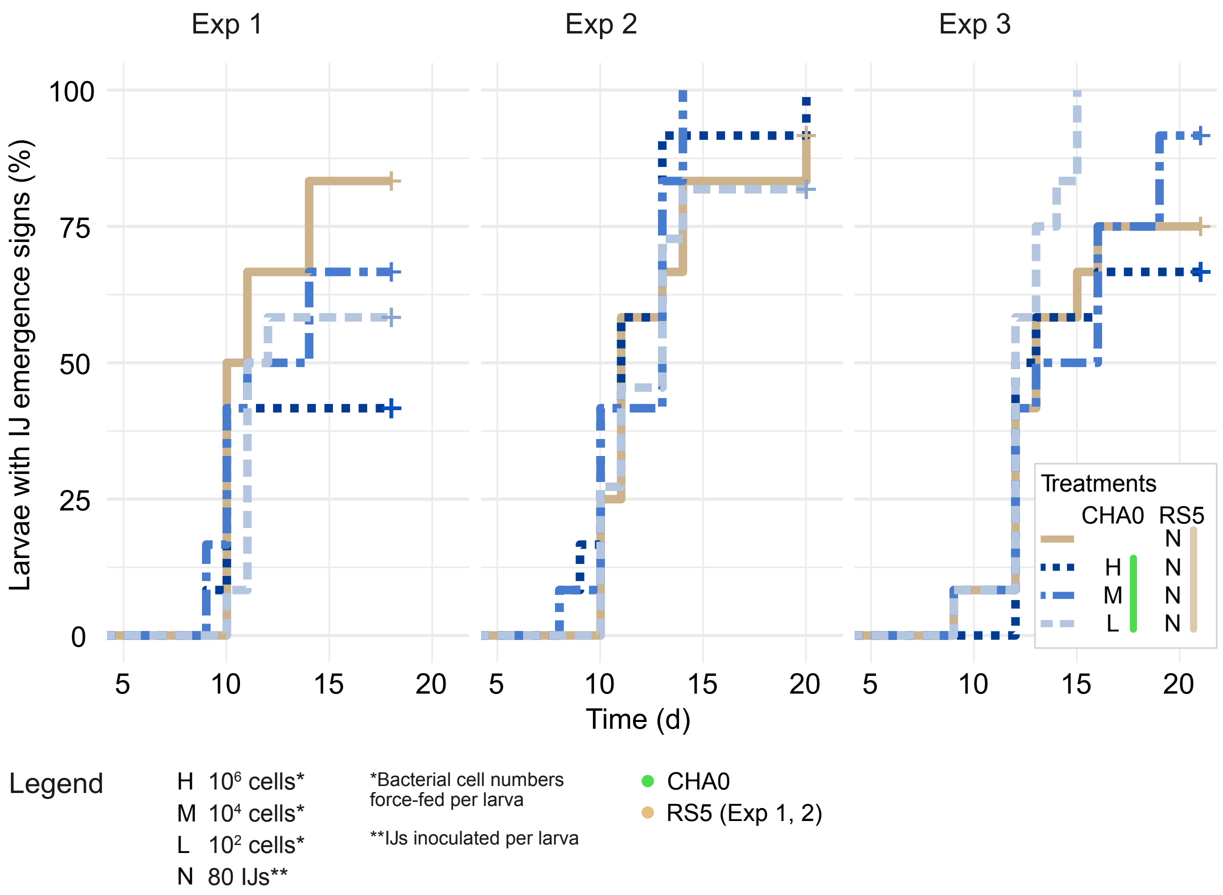

Fig. S5: **Infective juvenile (IJ) emergence time points for *S. feltiae* RS5 following natural infection.** *G. mellonella* larvae were force-fed with *P. protegens* CHA0 or LB followed by infection with the nematode *S. feltiae* RS5 associated with *X. bovienii* SM5. Dead, infected larvae were transferred to White traps and regularly checked for signs of IJ emergence from 8-21 dpi. Coloured lines and capital letters in the treatment box indicate which organisms (CHA0 = green, RS5 with SM5 = beige) and in which quantities (H = 10^6 cells, M = 10^4 cells, L = 10^2 cells, N = 80 IJs) were added to the respective treatments. Kaplan-Meier curves show the percentage of larvae with signs of IJ emergence. Three replicate experiments (N=3) are shown.

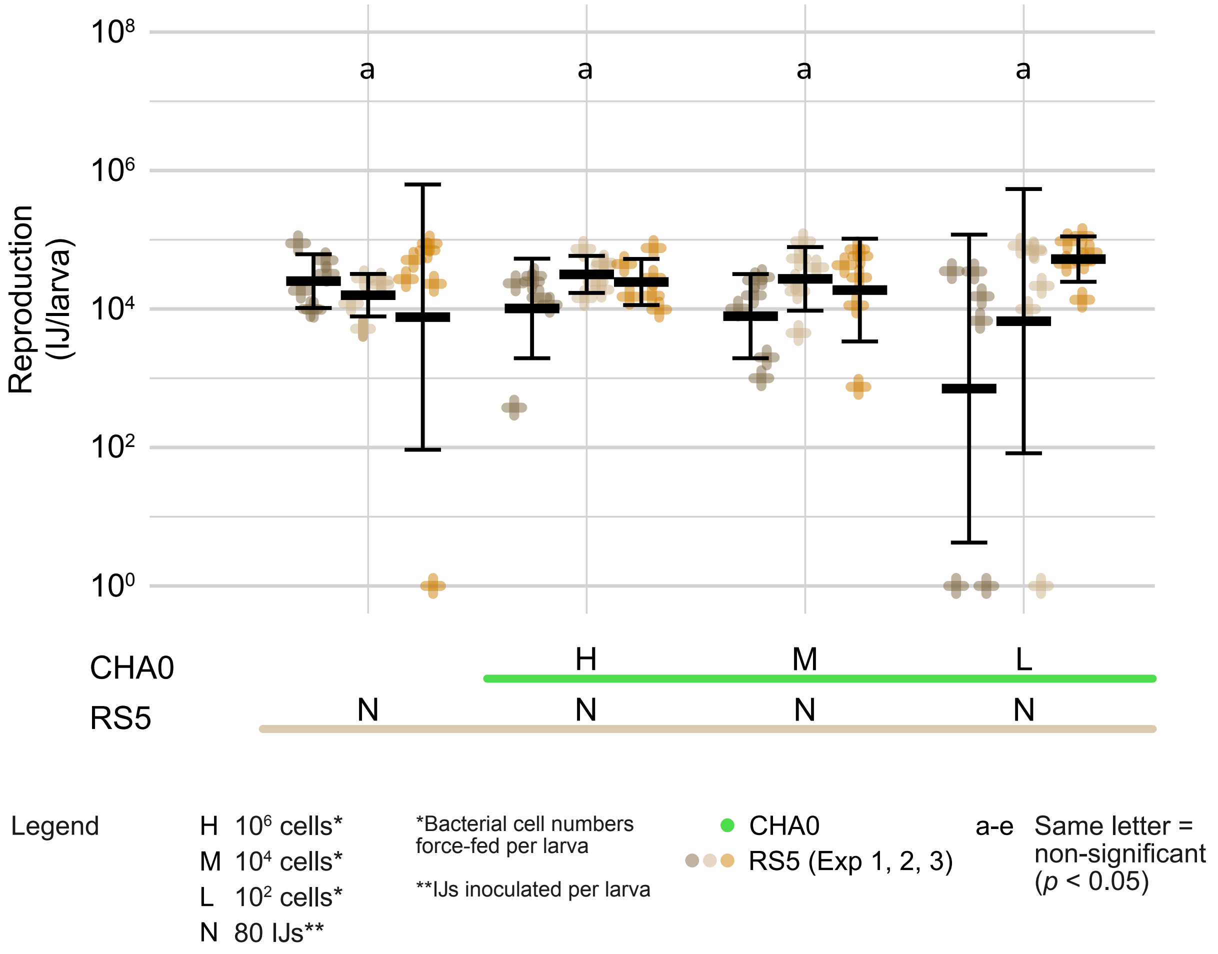

Fig. S6: **Infective juvenile (IJ) emergence numbers of *S. feltiae* RS5 following natural infection.** *G. mellonella* larvae were force-fed with *P. protegens* CHA0 or LB followed by infection with the nematode *S. feltiae* RS5 associated with *X. bovienii* SM5. Dead, infected larvae were transferred to White traps. Emerging IJs were collected until 21 dpi and then counted. Coloured lines and capital letters below the graphs indicate which organisms (CHA0 = green, RS5 with SM5 = beige) and in which quantities (H = 10^6 cells, M = 10^4 cells, L = 10^2 cells, N = 80 IJs) were added to the respective treatments. The numbers of emerged IJs per larva are shown as individual data points with mean and standard deviation. Three replicate experiments (N=3) are shown, and data were pooled for statistical analysis. Lower case letters (a-e) refer to significant differences between treatments according to a linear mixed effect model and post-hoc pairwise comparisons.

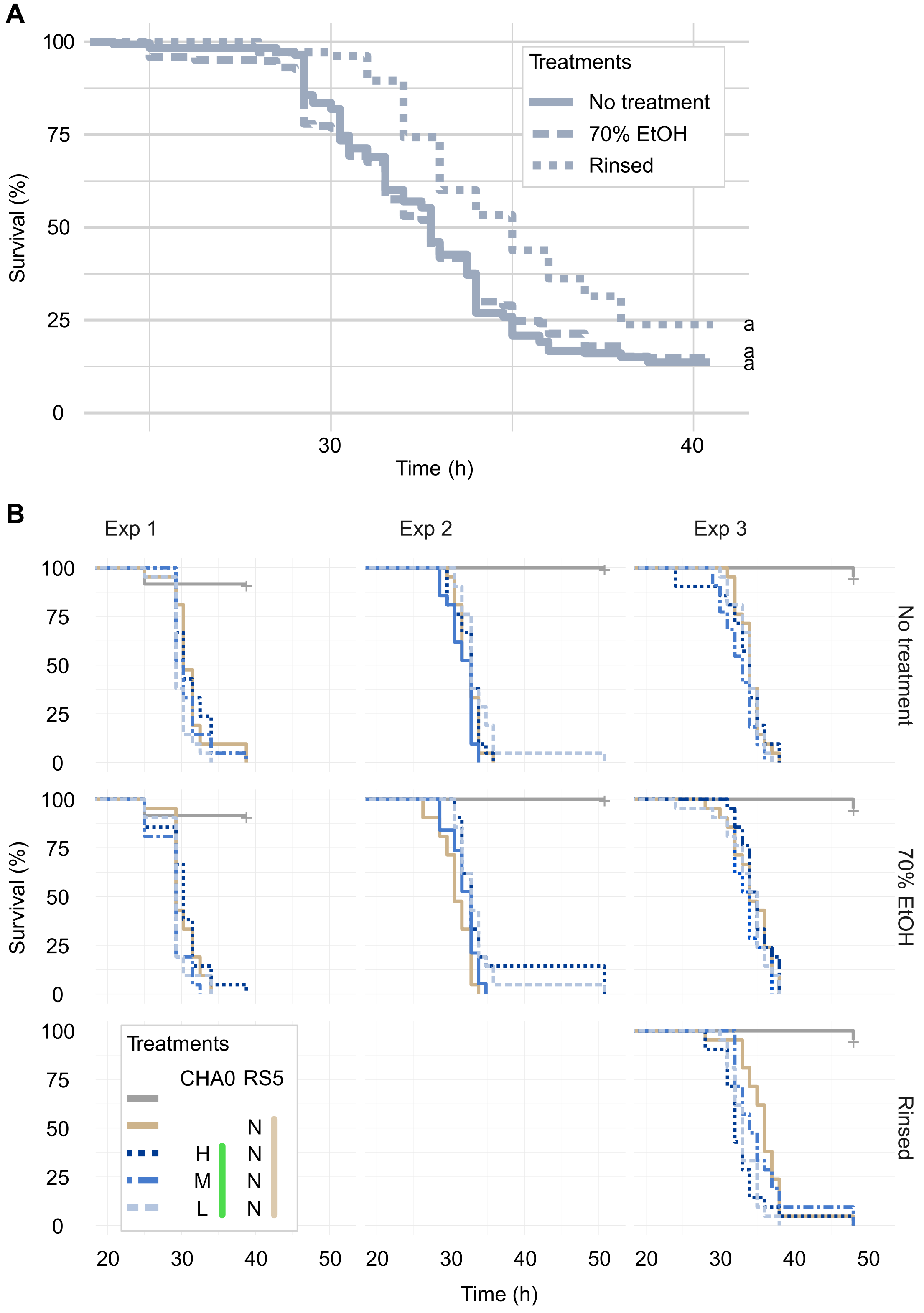

Legend

H 10^6 cells*
M 10^4 cells*
L 10^2 cells*
N 80 IJs**

*Bacterial cell numbers force-fed per larva

**IJs inoculated per larva

● CHA0
● RS5

a-e Same letter = non-significant ($p < 0.05$)

Fig. S7: **Virulence of emerged infective juveniles (IJs) of *S. feltiae* RS5 following natural infection.** *G. mellonella* larvae were force-fed with *P. protegens* CHA0 or LB followed by infection with the nematode *S. feltiae* RS5 associated with *X. bovienii* SM5. Dead, infected larvae were transferred to White traps and emerging IJs were collected, surface-disinfected, and their virulence was tested in *G. mellonella* infection assays. The effect of surface-disinfection (no treatment (untreated), rinsing with water (rinsed), surface-disinfection with 70% EtOH (70% EtOH)) on virulence was tested (**A**) and all surface-disinfection treatments vs. replicates are displayed (**B**). Coloured lines and capital letters in the treatment box indicate which organisms (CHA0 = green, RS5 with SM5 = beige) and in which quantities (H = 10^6 cells, M = 10^4 cells, L = 10^2 cells, N = 80 IJs) were added to the respective treatments. Kaplan-Meier curves show percentage of larval survival after exposure to emerged IJs. The experiment was repeated twice (N=3), and data were pooled for statistical analysis (A). Lowercase letters (a-e) refer to significant differences between treatments according to a Cox model and post-hoc pairwise comparisons (A).

A *P. protegens* CHA0

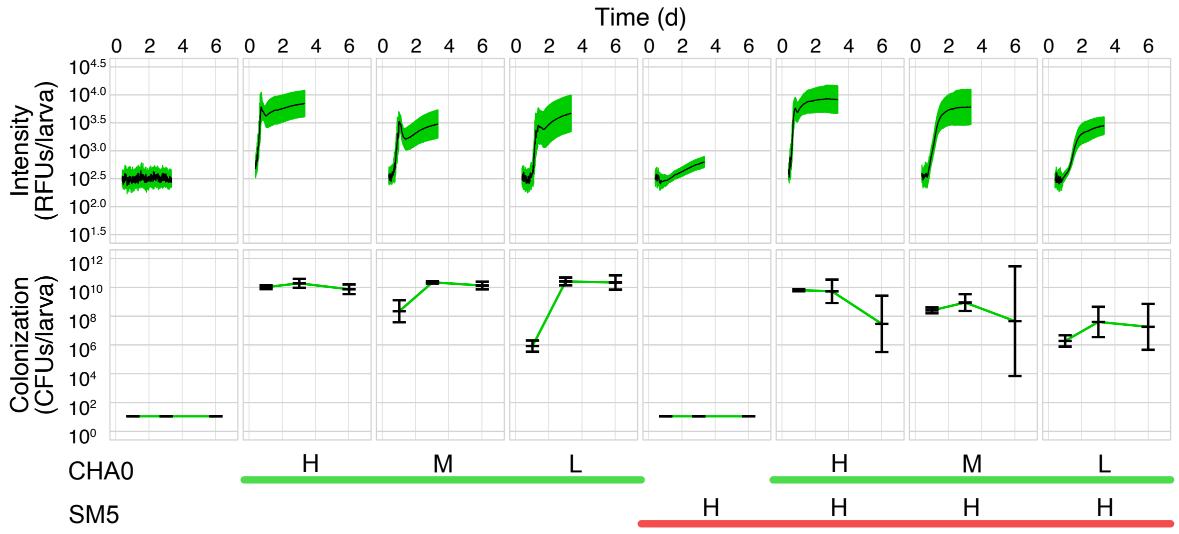

B *X. bovienii* SM5

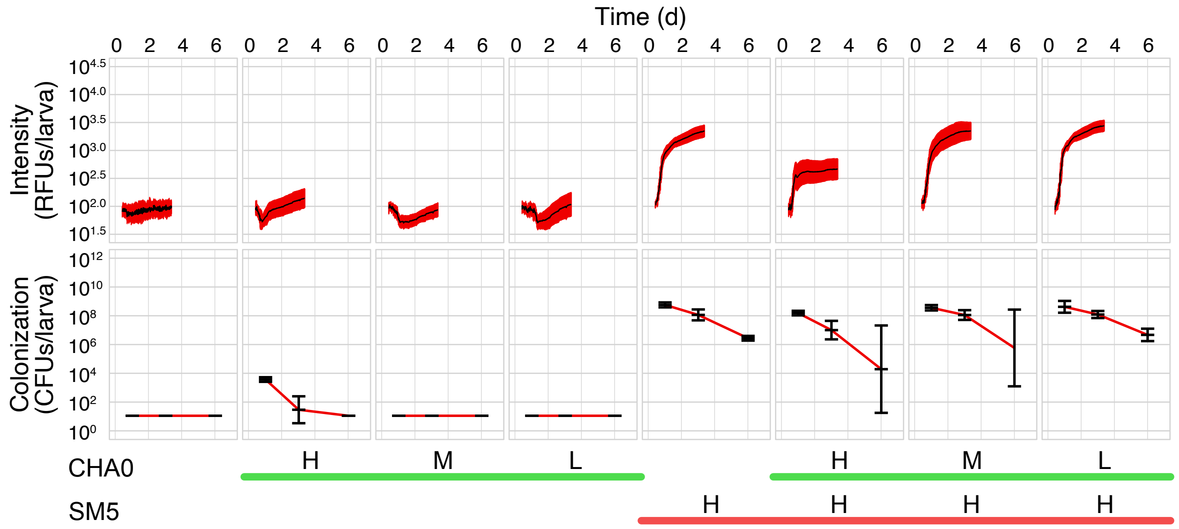

C Melanization and fluorescence phenotypes - 2 dpi

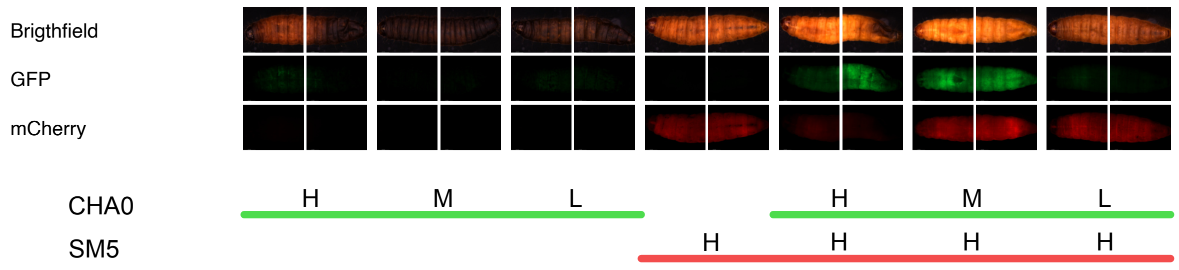

Legend

H 10^6 cells*
M 10^4 cells*
L 10^2 cells*

*Bacterial cell numbers injected per larva

● CHA0
● SM5

Fig. S8: **Comparison of selective plating (colonization) and fluorescence detection (intensity), two methods to study the interactions between *P. protegens* CHA0 and *X. bovienii* SM5 in the haemolymph of *G. mellonella* larvae: Experiment 2.**

The proliferation of *P. protegens* CHA0 **(A)** and *X. bovienii* SM5 **(B)** was monitored in single and combined treatments after haemolymph-injection into *G. mellonella* larvae. Besides selective plating, the fluorescence intensity emitted from GFP-tagged (CHA0) or mCherry-tagged (SM5) bacteria was measured through the larval body using a multimode microplate reader. This aimed at an indirect detection of the bacterial proliferation dynamics without destroying the carcass. Relative fluorescence units (RFUs) per larva were measured over time using filters for GFP or mCherry detection. Means are shown in black and standard deviations in green for CHA0 and red for SM5. Colony forming units (CFUs) per larva were determined by plating homogenized larvae on selective media at 1, 3, and 6 days post infection (dpi). Mean and standard deviation are shown. Images **(C)** show representative larvae at 2 dpi under brightfield, GFP, or mCherry conditions. Coloured lines and capital letters below the graphs indicate which organisms (CHA0 = green, SM5 = red) and in which quantities (H = 10^6 cells, M = 10^4 cells, L = 10^2 cells) were added to the respective treatments. The experiment was conducted twice (N=2). Experiment 1 is shown in Fig. 5.

A *P. protegens* CHA0

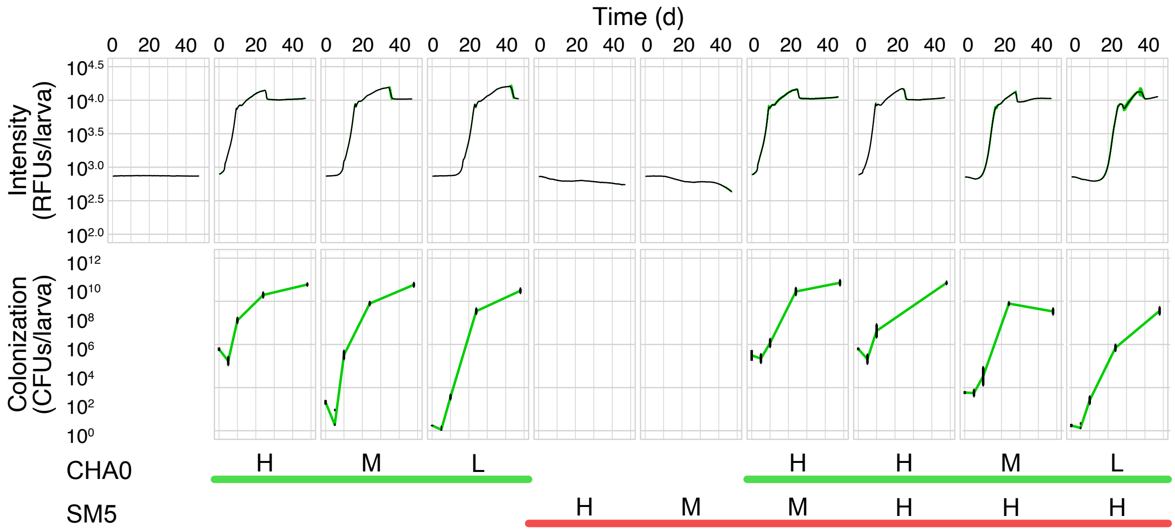

B *X. bovienii* SM5

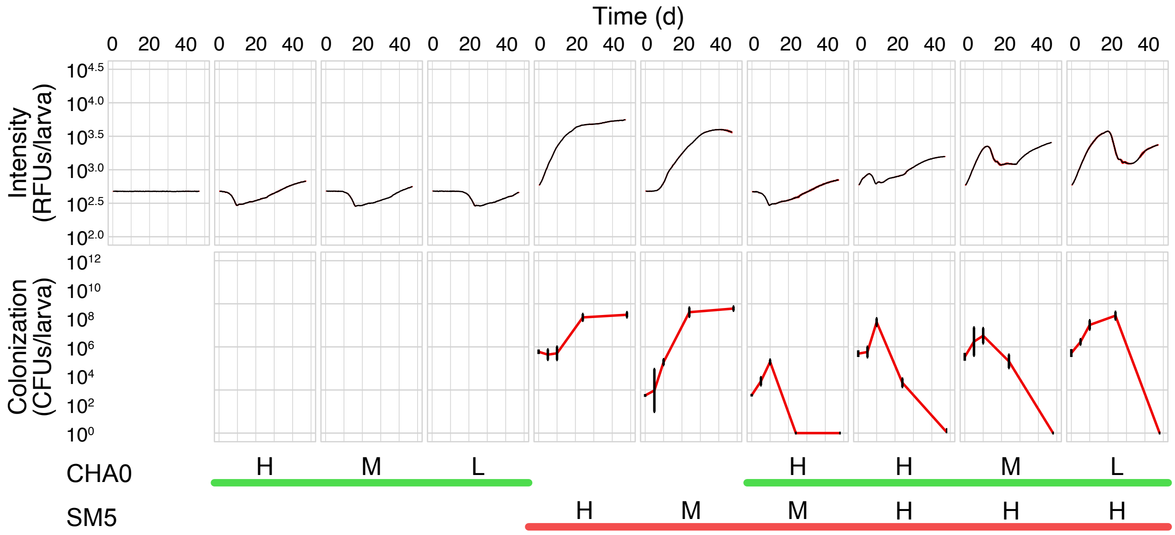

Legend

H 10^6 cells*
M 10^4 cells*
L 10^2 cells*

*Bacterial cell numbers inoculated in 100 µl liquid medium

● CHA0
● SM5

Fig. S9: **Comparison of selective plating (colonization) and fluorescence detection (intensity), two methods to study the interactions between *P. protegens* CHA0 and *X. bovienii* SM5 in vitro: Experiment 1.**

The proliferation of *P. protegens* CHA0 **(A)** and *X. bovienii* SM5 **(B)** was monitored in single and combined treatments after incubation in LB. Besides selective plating, the fluorescence intensity emitted from GFP-tagged (CHA0) or mCherry-tagged (SM5) bacteria was measured using a multimode microplate reader. This aimed at an indirect detection of the bacterial proliferation dynamics without destroying the insect. Relative fluorescence units (RFUs) per larva were measured over time using filters for GFP or mCherry detection. Means are shown in black and standard deviations in green for CHA0 and red for SM5. Colony forming units (CFUs) per larva were determined by plating cultures on selective media at 1, 3, and 6 days post infection (dpi). Mean and standard deviation are shown. Coloured lines and capital letters below the graphs indicate which organisms (CHA0 = green, SM5 = red) and in which quantities (H = 10^6 cells, M = 10^4 cells, L = 10^2 cells) were added to the respective treatments. The experiment was conducted twice (N=2), experiment 2 is shown in Fig. S10.

A *P. protegens* CHA0

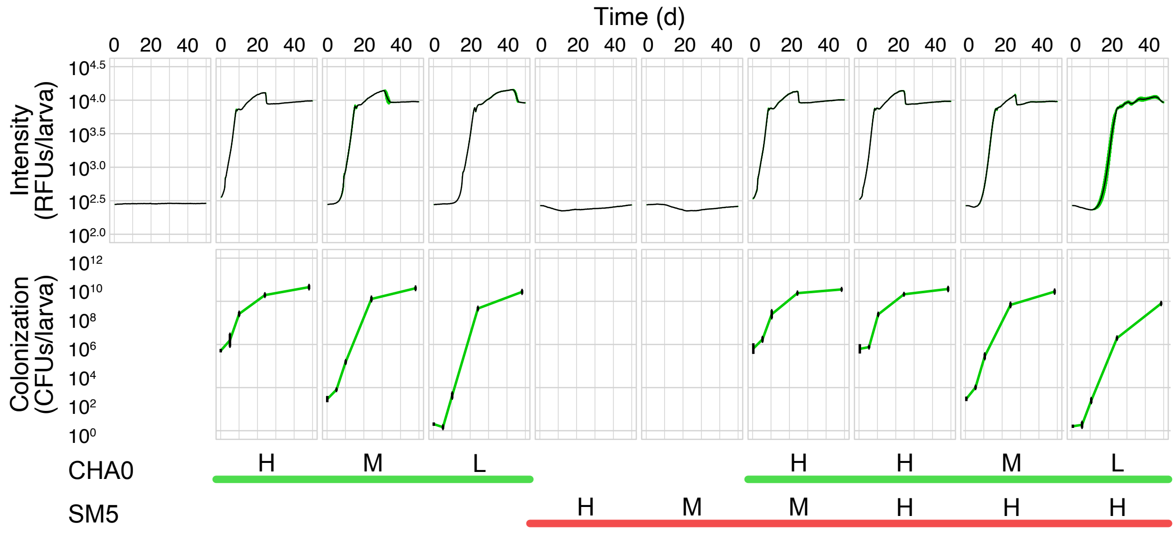

B *X. bovienii* SM5

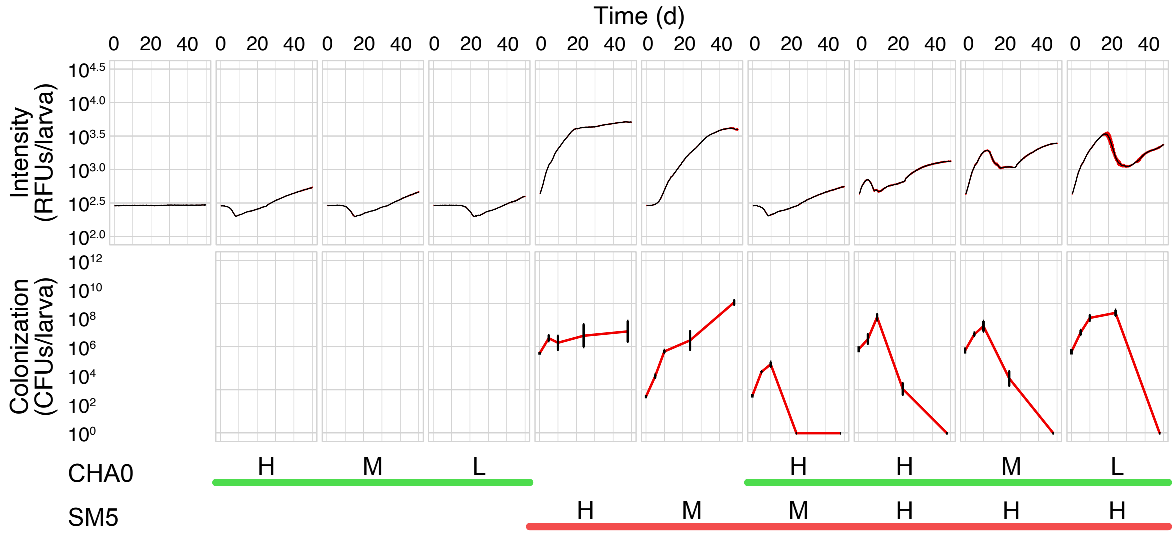

Legend

H 10^6 cells*
M 10^4 cells*
L 10^2 cells*

*Bacterial cell numbers inoculated in 100 µl liquid medium

● CHA0
● SM5

Fig. S10: **Comparison of selective plating (colonization) and fluorescence detection (intensity), two methods to study the interactions between *P. protegens* CHA0 and *X. bovienii* SM5 in vitro: Experiment 2.**
The proliferation of *P. protegens* CHA0 **(A)** and *X. bovienii* SM5 **(B)** was monitored in single and combined treatments after incubation in LB. Besides selective plating, the fluorescence intensity emitted from GFP-tagged (CHA0) or mCherry-tagged (SM5) bacteria was measured using a multimode microplate reader. This aimed at an indirect detection of the bacterial proliferation dynamics without destroying the insect. Relative fluorescence units (RFUs) per larva were measured over time using filters for GFP or mCherry detection. Means are shown in black and standard deviations in green for CHA0 and red for SM5. Colony forming units (CFUs) per larva were determined by plating cultures on selective media at 1, 3, and 6 days post infection (dpi). Mean and standard deviation are shown. Coloured lines and capital letters below the graphs indicate which organisms (CHA0 = green, SM5 = red) and in which quantities (H = 10^6 cells, M = 10^4 cells, L = 10^2 cells) were added to the respective treatments. The experiment was conducted twice (N=2), experiment 1 is shown in Fig. S9.

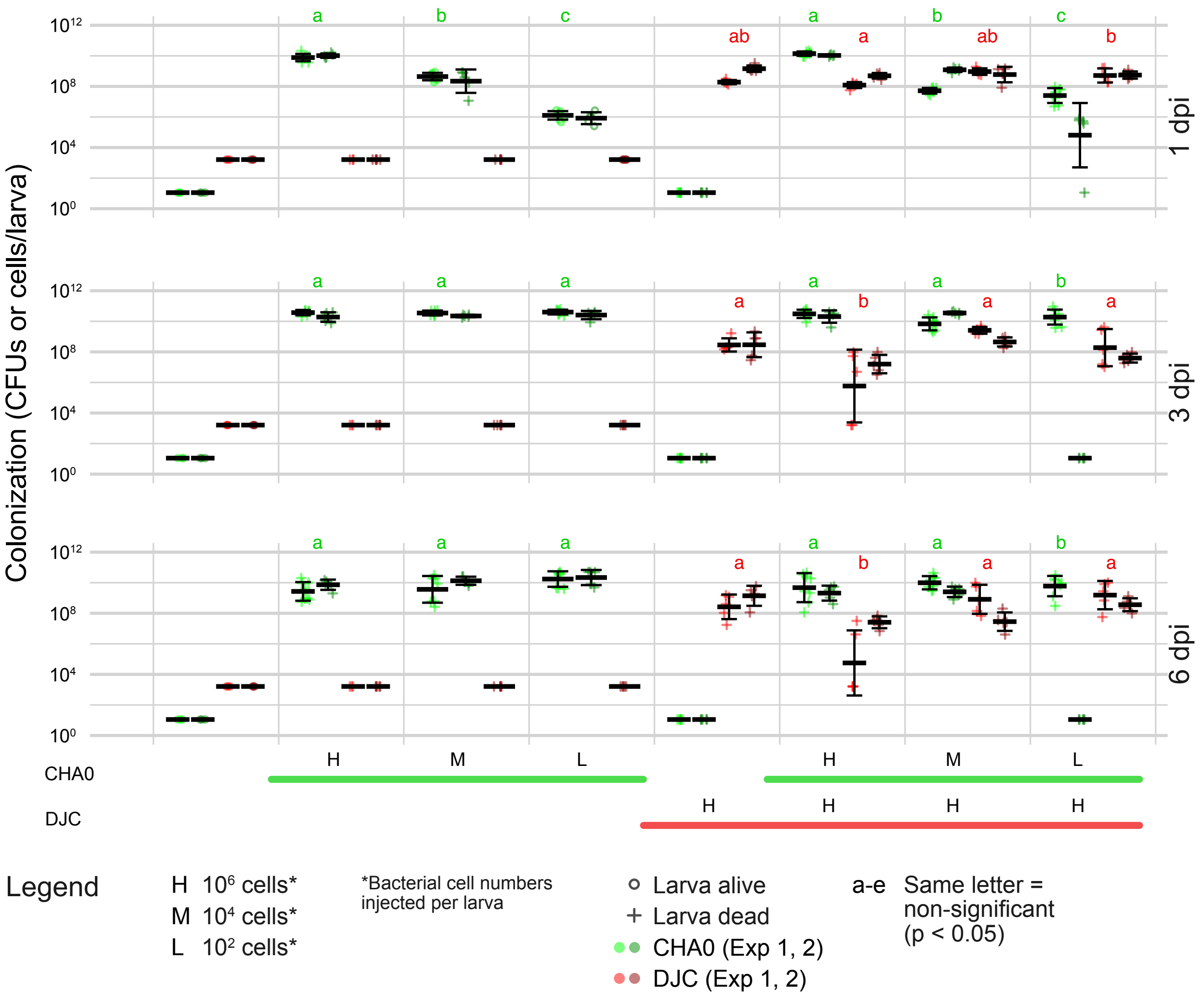

Fig. S11: **Interactions between *P. protegens* CHA0 and *P. laumondii* DJC in *G. mellonella* larvae following haemolymph-injection.**

The proliferation of *P. protegens* CHA0 and *P. laumondii* DJC was monitored in single and combined treatments after haemolymph-injection into *G. mellonella* larvae. Colony forming units (CFUs) per larva were determined by plating homogenized larvae on selective media. Individual data points are shown in green for CHA0 and red for DJC with mean and standard deviation. Coloured lines and capital letters below the graphs indicate which organisms (CHA0 = green, DJC = red) and in which quantities (H = 10^6 cells, M = 10^4 cells, L = 10^2 cells) were added to the respective treatment. Two replicate experiments (N=2) are shown, and data were pooled for statistical analysis. Lowercase letters (a-e) refer to significant differences between treatments according to a linear mixed effect model and post-hoc pairwise comparison and can be compared when written in the same colour.

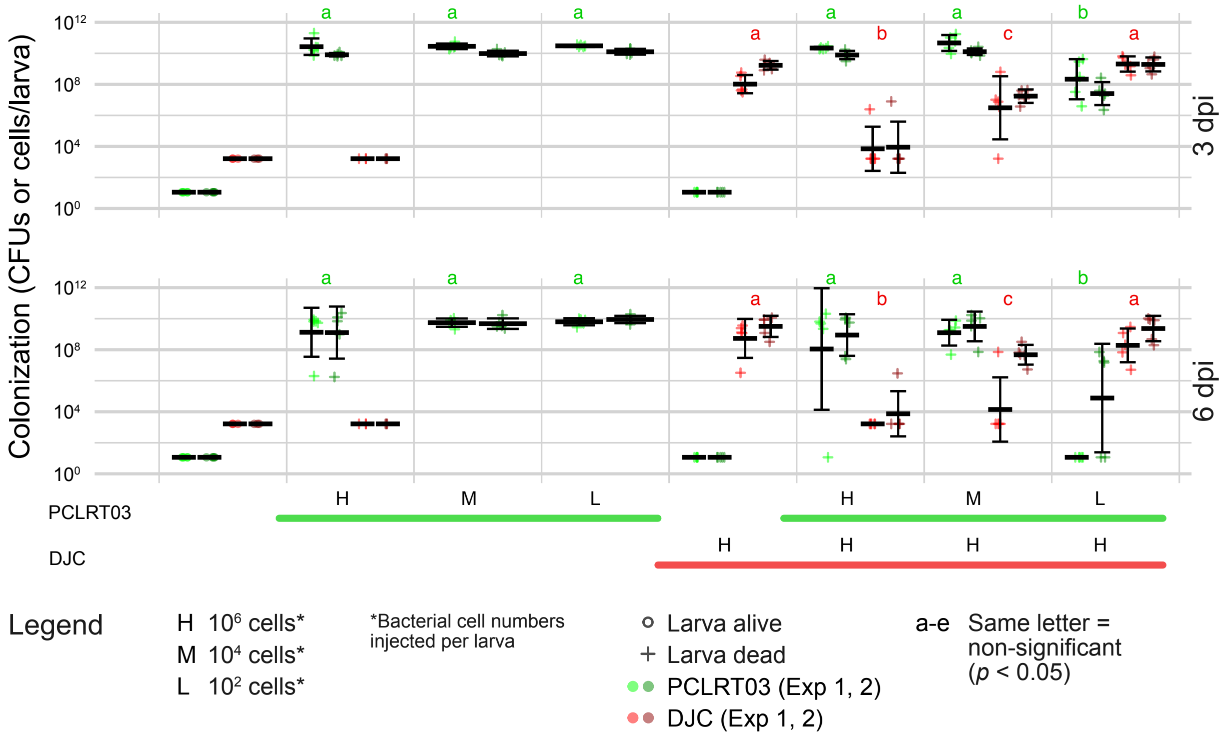

Fig. S12: **Interactions between *P. chlororaphis* PCLRT03 and *P. laumondii* DJC in *G. mellonella* larvae following haemolymph-injection.**

The proliferation of *P. chlororaphis* PCLRT03 and *P. laumondii* DJC was monitored in single and combined treatments after haemolymph-injection into *G. mellonella* larvae. Colony forming units (CFUs) per larva were determined by plating homogenized larvae on selective media. Individual data points are shown in green for PCLRT03 and red for DJC with mean and standard deviation. Coloured lines and capital letters below the graphs indicate which organisms (PCLRT03 = green, DJC = red) and in which quantities (H = 10^6 cells, M = 10^4 cells, L = 10^2 cells) were added to the respective treatment. Two replicate experiments (N=2) are shown, and data were pooled for statistical analysis. Lowercase letters (a-e) refer to significant differences between treatments according to a linear mixed effect model and post-hoc pairwise comparison and can be compared when written in the same colour.

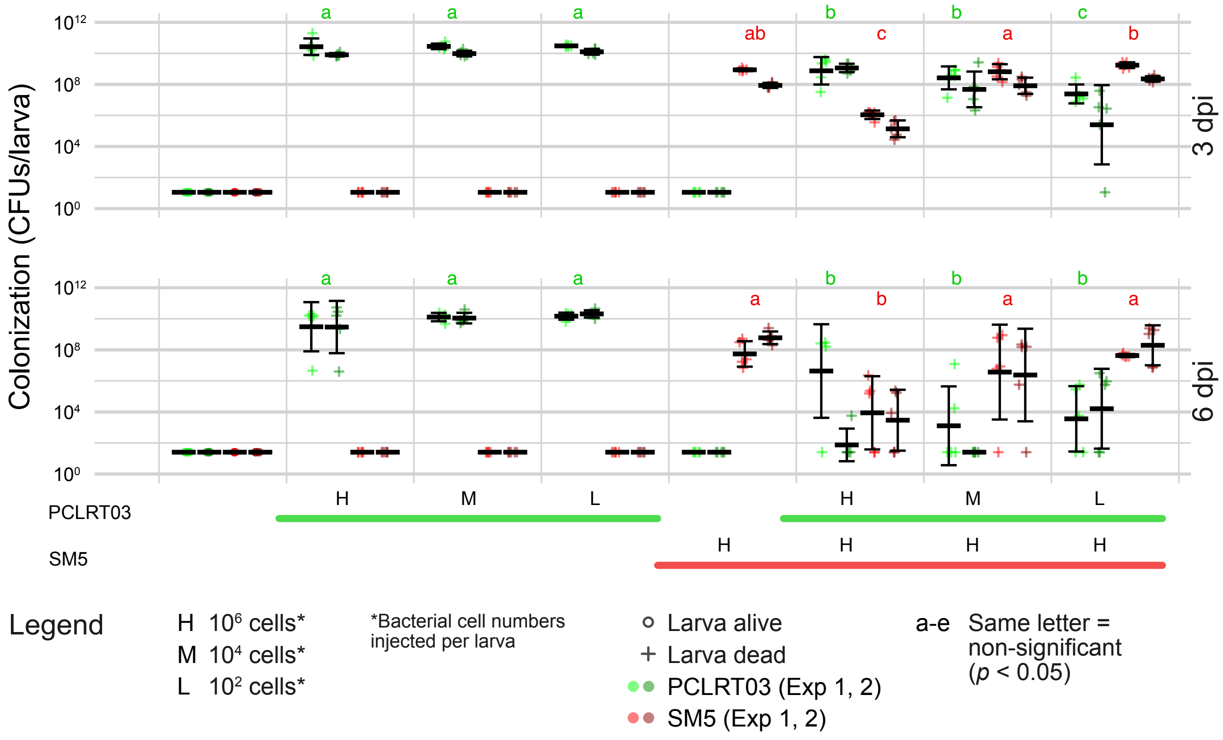

Fig. S13: **Interactions between *P. chlororaphis* PCLRT03 and *X. bovienii* SM5 in *G. mellonella* larvae following haemolymph-injection.**

The proliferation of *P. chlororaphis* PCLRT03 and *X. bovienii* SM5 was monitored in single and combined treatments after haemolymph-injection into *G. mellonella* larvae. Colony forming units (CFUs) per larva were determined by plating homogenized larvae on selective media. Individual data points are shown in green for PCLRT03 and red for SM5 with mean and standard deviation. Coloured lines and capital letters below the graphs indicate which organisms (PCLRT03 = green, SM5 = red) and in which quantities (H = 10^6 cells, M = 10^4 cells, L = 10^2 cells) were added to the respective treatment. Two replicate experiments (N=2) are shown, and data were pooled for statistical analysis. Lowercase letters (a-e) refer to significant differences between treatments according to a linear mixed effect model and post-hoc pairwise comparison and can be compared when written in the same colour.

References

1. King EO, Ward MK, Raney DE. Two simple media for the demonstration of pyocyanin and fluorescin. *J Lab Clin Med* 1954; **44**: 301–7.

2. Bertani G. Studies on lysogenesis I: the mode of phage liberation by lysogenic *Escherichia coli*. *J Bacteriol* 1951; **62**: 293–300.

3. Vicente-Díez I, Blanco-Pérez R, Chelkha M et al. Exploring the use of entomopathogenic nematodes and the natural products derived from their Symbiotic bacteria to control the grapevine moth, *Lobesia botrana* (Lepidoptera: Tortricidae). *Insects* 2021; **12**: 1033.

4. Vesga P, Flury P, Vacheron J et al. Transcriptome plasticity underlying plant root colonization and insect invasion by *Pseudomonas protegens*. *ISME J* 2020; **14**: 2766–82.

5. Spescha A, Zwyssig M, Hess Hermida M et al. When competitors join forces: Consortia of entomopathogenic microorganisms increase killing speed and mortality in leaf- and root-feeding insect hosts. *Microb Ecol* 2023; **86**: 1947-60.

6. Regaiolo A, Dominelli N, Andresen K, Heermann R. The biocontrol agent and insect pathogen *Photorhabdus luminescens* interacts with plant roots. *Appl Environ Microbiol* 2020; **86**: e00891-20.
